# Segregation of sister chromosomes during the shape change of developing *Myxococcus xanthus* cells

**DOI:** 10.1128/jb.00328-25

**Published:** 2025-09-26

**Authors:** Y. Hoang, Yann S. Dufour, Lee Kroos

**Affiliations:** 1 Department of Biochemistry and Molecular Biology, Michigan State University 3078, East Lansing, Michigan, USA; 2 Department of Microbiology, Genetics, & Immunology, Michigan State University 3078, East Lansing, Michigan, USA; The Ohio State University, Columbus, Ohio, USA

**Keywords:** sister chromosomes, nucleoid, cell shape change, sporulation, bacterial development, *Myxococcus xanthus*, chromosome segregation

## Abstract

Chromosome organization is critical for the maintenance of genetic integrity. Most studies of bacterial nucleoids have focused on growing rod-shaped organisms. Studying nucleoid dynamics during *Myxococcus xanthus* development offers the unique opportunity to investigate the localization of two sister chromosomes as rod-shaped cells transition into round spores. During starvation-induced multicellular development, DNA replication is required for *M. xanthus* rods to transition into spores with two copies of the chromosome. Here, we report novel approaches using confocal fluorescence microscopy to observe the chromosome number and arrangement, and nucleoid localization in developing cells *in situ*. We discovered that sister chromosomes are present in some rods and transitioning cells (TCs) early in development. The arrangement of the two chromosomes in developing cells was novel compared to predivisional growing cells studied previously. We observed segregated nucleoids in ~40% of TCs and spores. The majority of TCs contained a crescent-shaped nucleoid along one side, perhaps due to ongoing chromosome segregation, whereas most spores appeared to have undergone nucleoid decondensation. During unicellular glycerol-induced sporulation of *M. xanthus*, we observed segregated nucleoids in only ~10%–20% of TCs and spores. In addition, early in starvation-induced development, we discovered a subpopulation of cells that may be spheroplasts destined for lysis, which is the fate of most cells under these conditions. Chromosome segregation in developing *M. xanthus* may be a bet-hedging strategy to increase survival under different conditions and/or an evolutionary remnant of ancestral events that included cell division to produce spores with one copy of the chromosome.

**IMPORTANCE:**

The cell cycle normally involves DNA replication, chromosome segregation, and cell division. During starvation-induced *Myxococcus xanthus* development, DNA replication is necessary for progression to spore formation, which occurs without cell division, resulting in spores with two copies of the chromosome. The organization of sister chromosomes during the morphological change of rod-shaped cells into round spores was unknown. We discovered that the two nucleoids often segregate during the transition from rods to spores. Mature spores contained decondensed nucleoids. Our observations raise important questions about the mechanism of chromosome segregation during *M. xanthus* development and the reason for its existence. We also discovered a subpopulation of developing cells with characteristics suggesting they are spheroplasts on the verge of cell death.

## INTRODUCTION

Spatial organization is a critical feature of all living systems. Although bacteria are the smallest form of cellular life, they still possess complex intracellular organization, such as polar localization of chemoreceptors (1), protein oscillations (2), and nucleoid organization (3–5). In terms of nucleoid organization, the genomic DNA must be compacted several orders of magnitude since the bacterial chromosome is ~1,000 times longer than the micrometer-sized cell. Chromosome organization is accomplished by both physical and biochemical factors, such as DNA supercoiling (6) and nucleoid-associated proteins (7, 8). The organization of the two nucleoids after DNA replication facilitates segregation of chromosomes before cell division. Proper chromosome segregation is essential to preserve the integrity of the genetic material for the continuation of the species. With the advancement of fluorescence microscopy and live-cell imaging, our knowledge of nucleoid condensation and segregation has increased. However, most studies of bacterial nucleoids have focused on the rod-shaped or crescent-shaped model organisms *Escherichia coli* (9), *Bacillus subtilis* (10), and *Caulobacter crescentus* (11) (reviewed in reference [12]). In this work, we studied nucleoid dynamics during the multicellular development of *Myxococcus xanthus* (reviewed in reference [13]), which offers the unique opportunity to investigate sister chromosome segregation as rod-shaped cells are converted into round spores.

The starvation-induced development of *M. xanthus* requires the coordinated movements of ~10^5^ cells to form a dome-shaped mound. Large populations can be induced to form many mounds of similar width and height. A majority of the population undergoes lysis during this process (14, 15). Some rod-shaped cells persist outside of mounds as peripheral rods (16). The temporal and spatial distribution of cells transitioning from rods to spores inside mounds or so-called “nascent fruiting bodies” (NFBs) has been described (17). There are more transitioning cells (TCs) and spores near the radial center of NFBs than near their perimeter. The TCs undergo peptidoglycan remodeling and adopt different shapes that may depend on their surroundings and/or non-uniformity in the remodeling process. DNA replication is tightly regulated to ensure that all spores have two copies of the chromosome (18). During vegetative growth, cells contain one or two copies of the genome, but the chromosome copy number drops to one when cells enter the stationary phase (18). As starving cells begin to form mounds, DNA replication is required for development to progress (19). Inhibition of DNA replication delays the developmental program, which can resume if the inhibition is removed (20). If a DNA replication inhibitor is added after mounds have formed, development proceeds (19). Fruiting body-derived spores contain two copies of the chromosome with the replication origin (*ori*) and terminus (*ter*) regions localized to the periphery of the spore (18).

*M. xanthus* can also form spores in response to certain chemicals. In contrast to starvation-induced development, glycerol-induced sporulation is much faster and is unicellular (i.e., cells do not form mounds by moving on a solid surface). The chromosome copy number varies in glycerol-induced spores, suggesting that DNA replication is less tightly regulated (18). Cells appear to bypass the DNA replication checkpoint and proceed to shape change.

Analysis of *M. xanthus* chromosome and replisome dynamics during growth and division revealed a novel arrangement of sister chromosomes in predivisional cells, with the *ori* regions ~ 1 µm from each cell pole and the *ter* regions near midcell (21). After division, the *ori* and *ter* regions were ~1 µm from the opposite poles of newborn cells. The chromosome arrangement was not examined in developing cells.

In this study, we investigated the chromosome number and arrangement, nucleoid localization, and cell shape of developing *M. xanthus* cells *in situ* using several confocal fluorescence microscopy methods. We found that two copies of the chromosome are present in some rods and TCs early in development, as well as in spores later, and the chromosome arrangement differs in developing cells compared to the *ori-ter-ter-ori* arrangement in predivisional cells during growth (21). We also discovered that the two nucleoids are segregated in ~40% of TCs and spores within NFBs, whereas only ~10%–20% of glycerol-induced spores exhibit segregated nucleoids. These findings raise many interesting questions about how and why chromosome segregation occurs during the shape change of developing *M. xanthus* cells. We also found a subpopulation of cells within early NFBs whose characteristics suggest they may be spheroplasts at a late stage of a dying process that ends with lysis, the fate of most cells during starvation-induced *M. xanthus* development (14, 15).

## RESULTS

### Sister chromosomes are present in some developing rods and TCs, as well as in spores

In a previous study, starvation-induced spores were dispersed from fruiting bodies, and flow cytometry showed that spores contain two copies of the chromosome (18). We used a fluorescent repressor operator system (22) and confocal laser scanning microscopy to visualize the chromosome number and arrangement in developing cells within NFBs. *M. xanthus* strains with *tetO* operator arrays inserted at specific chromosomal loci were described previously (21). In that study, TetR-YFP was produced under the control of a copper-inducible promoter during growth. However, copper interferes with starvation-induced submerged culture development (E. Titus and L. Kroos, unpublished data). Therefore, we constructed strains to produce TetR-YFP under the control of an IPTG-inducible promoter in backgrounds with *tetO-*arrays inserted at 33° and 270° from the origin of replication (*ori*), resulting in *M. xanthus* strains YH3 and YH4, respectively (Fig. S1A). We used *tetO* arrays inserted at different chromosomal loci to detect potential bias in the arrangement of sister chromosomes. IPTG addition to induce TetR-YFP production for binding to *tetO-*arrays did not affect the growth of YH3 or YH4, or wild-type control strain DK1622 (Fig. S1B).

**Fig 1:**
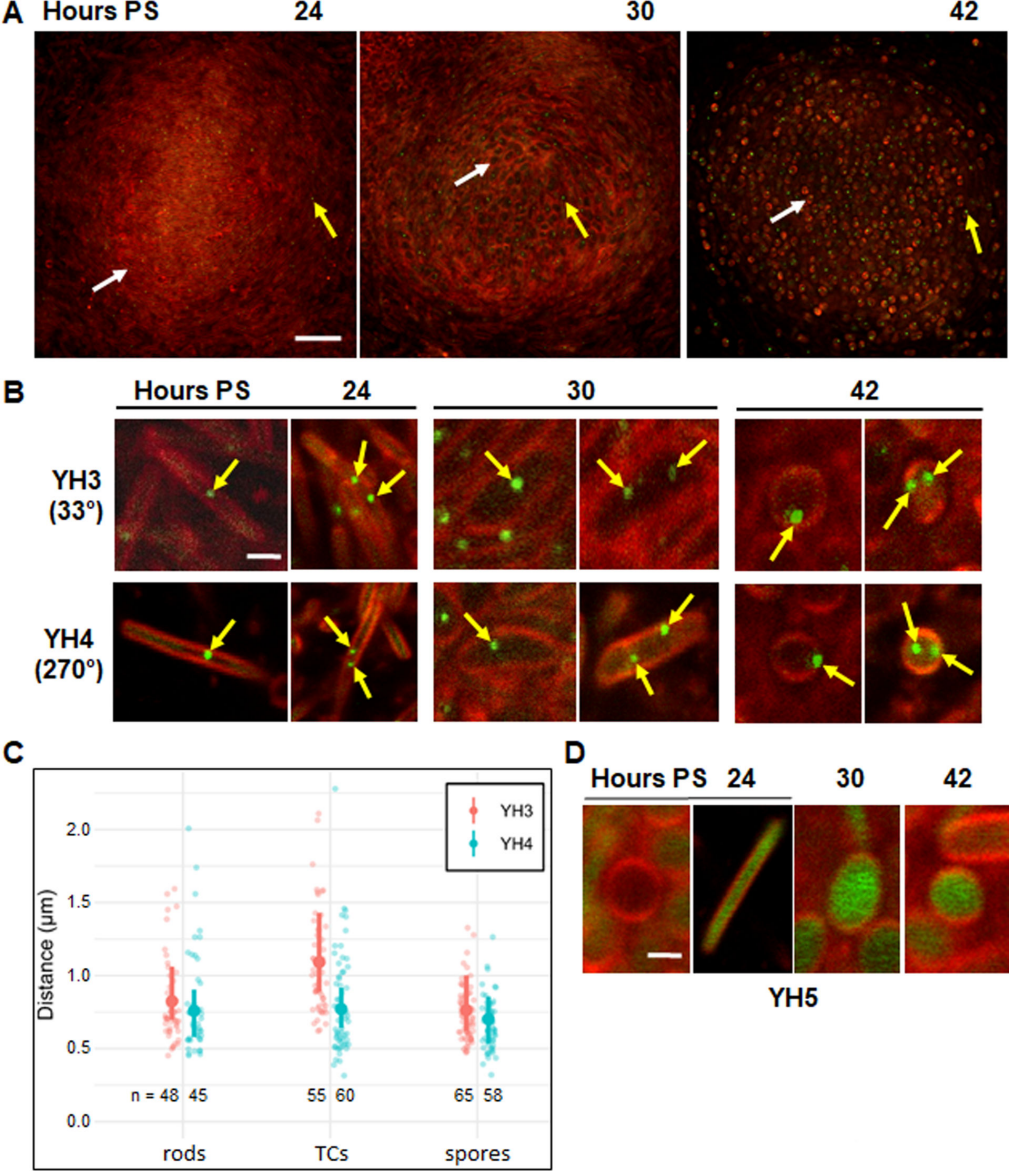
Sister chromosomes are present early in development in some rods and TCs, as well as later in spores. (**A**) Visualization of chromosomes using a fluorescent repressor operator system during development. *M. xanthus* strains YH3, YH4, and YH5 were starved under submerged culture conditions. IPTG (1 mM) and FM 4-64 (5 µg/mL) were added at the start of starvation. Confocal images were acquired at the indicated times PS and merged to show both TetR-YFP (yellow-green) and FM 4-64 staining of the cell membrane (red). Images of YH3 show an optical section near the base of the same NFB over time. White arrows indicate a rod-shaped cell at 24 h, a TC at 30 h, and a spore at 42 h. Yellow arrows point to TetR-YFP foci. Bar, 20 µm. See Fig. S2 for images of YH4 and YH5. (**B**) Enlarged images of representative YH3 and YH4 cells. Images show rods at 24 h, TCs at 30 h, and spores at 42 h with one or two TetR-YFP foci (arrows). The position of the *tetO-*array relative to *ori* is shown in parentheses. Bar, 1 µm. (**C**) Distance between TetR-YFP foci. The distance was measured for the indicated number of rods at 24 h, TCs at 30 h, and spores at 42 h from five biological replicates. Small dots, individual cells. Large dot, median. Vertical line, 95% credible interval. (**D**) Enlarged images of representative YH5 cells lacking a *tetO-*array. Images show a round cell lacking TetR-YFP fluorescence at 24 h (leftmost panel), and a rod-shaped cell, a TC, and a spore exhibiting cytoplasmic TetR-YFP fluorescence at 24, 30, and 42 h, respectively. Bar, 1 µm.

Upon starvation in submerged culture, YH3 and YH4 formed mounds by 24 h, and most cells near the base were rod-shaped (Fig. 1A; Fig. S2A), as expected (17). In the same NFBs, we observed rods and TCs at 30 h poststarvation (PS). By 42 h, most cells had become round spores. We also saw one or two yellow-green fluorescent foci in some cells, which were due to localized binding of TetR-YFP to the *tetO*-array, since the control strain YH5 lacking a *tetO*-array showed no TetR-YFP foci and instead showed TetR-YFP fluorescence distributed throughout the cytoplasm of most developing cells (Fig. S2B). Figure 1B shows representative rods, TCs, and spores of YH3 and YH4 that exhibited one TetR-YFP focus (left panels) or two foci (right panels). The cells with two TetR-YFP foci demonstrate the presence of two copies of the chromosome. We conclude that sister chromosomes are present early in development in some rods and TCs, as well as later in spores (18).

The developing YH3 and YH4 cells with two TetR-YFP foci provided an opportunity to investigate the arrangement of sister chromosomes. By examining an optical section near the base of ≥30 different NFBs for each strain at different times PS, we were able to measure the distance between two TetR-YFP foci for ≥45 rods, TCs, and spores (Fig. 1C), as well as the cell dimensions based on membrane staining (Fig. S3). The dimensions of the cells examined were similar for the two strains. The distance between TetR-YFP foci correlated positively with the length of rods and TCs, and with the diameter of spores (Fig. S4). Although individual cells can have different orientations relative to the optical section, we reasoned that significant differences between YH3 and YH4 in terms of the distance between TetR-YFP foci would suggest a biased arrangement of sister chromosomes. However, taking into account the effect of cell length on the distance between TetR-YFP foci (Fig. S4), our data do not support a biased arrangement of the two chromosomes in rods, TCs, or spores (Fig. 1C). Interestingly, the distance between foci in developing cells is less than in predivisional cells during vegetative growth, as determined using strains with *tetO-*arrays located at 33° or 270° from *ori* (21) (Fig. S1C). Hence, the arrangement of sister chromosomes differs between predivisional and developing cells (see Discussion).

No TetR-YFP foci were evident in some developing YH3 and YH4 cells (Fig. 1A; Fig. S2A). In these cells, one or more TetR-YFP foci might be present, but not in the 0.5 µm optical section captured in the image, since cells in NFBs are at various orientations relative to the optical section and most dimensions of cells exceed 0.5 µm (Fig. S3). Alternatively, TetR-YFP expression may be insufficient in some cells to create a detectable focus. As a control, we induced TetR-YFP expression in a growing culture of YH4 and imaged vegetative cells in a monolayer between an agarose pad and a glass cover slip. We observed many cells with one TetR-YFP focus of variable intensity and many cells with no detectable focus (Fig. S5). Very few cells showed two foci, and adjusting the optical section up and down did not reveal many additional foci. We conclude that both insufficient TetR-YFP expression and the thinness of the optical section likely contribute to the apparent absence of foci in some developing cells.

YH5 lacking a *tetO-*array failed to show TetR-YFP foci and instead showed TetR-YFP fluorescence distributed throughout the cytoplasm of most rods, TCs, and spores (Fig. 1D; Fig. S2B). However, we noticed some round cells with no cytoplasmic fluorescence at 24 h PS (Fig. 1D, leftmost panel; Fig. S2B, blue arrow). We speculate that these cells cannot maintain the level or proper folding of TetR-YFP. YH3 and YH4 also exhibited round cells with no apparent cytoplasmic fluorescence at 24 h (Fig. 1A; Fig. S2A). Based on additional characterization presented below, these cells appear to be spheroplasts destined for lysis, the fate of most starving developmental cells (14, 15).

### Evidence for segregated nucleoids in some TCs and spores

**Fig 2:**
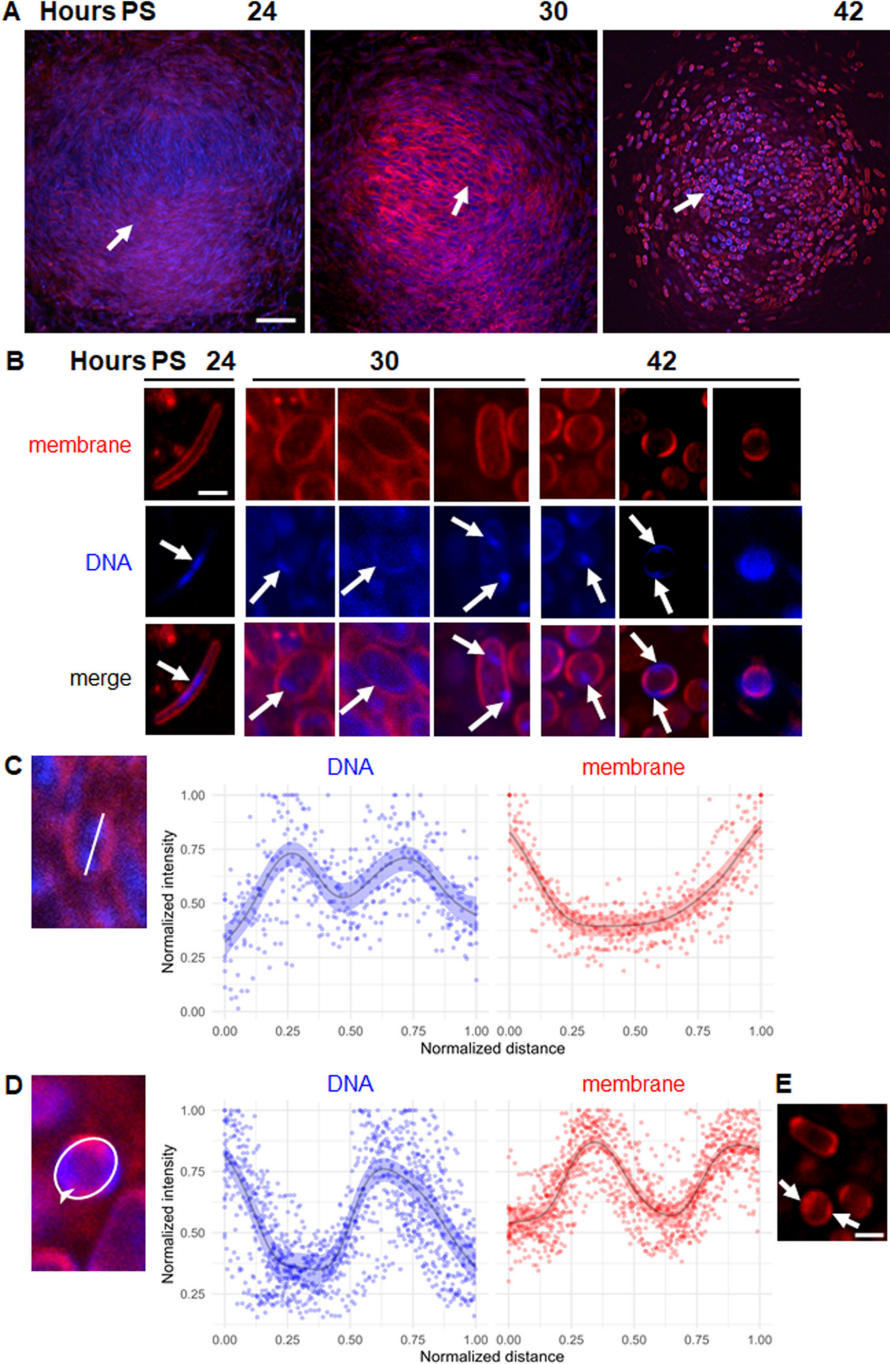
Evidence for segregated nucleoids in some TCs and spores. (**A**) Visualization of DAPI-stained DNA during development. *M. xanthus* wild-type strain DK1622 was starved under submerged culture conditions. FM 4-64 (5 µg/mL) was added at the start of starvation. DAPI (10 µg/mL) was added 30 min before imaging. Confocal images were acquired at the indicated times PS to show DAPI staining of DNA (blue) and FM 4-64 staining of the cell membrane (red). Images show an optical section near the base of representative NFBs, with the red and blue channels merged. Arrows indicate a rod-shaped cell at 24 h, a TC at 30 h, and a spore at 42 h. Bar, 20 µm. (**B**) Enlarged images of representative cells. Images show a rod at 24 h, TCs at 30 h, and spores at 42 h with one or two DAPI-stained DNA fluorescent patches (arrows). Bar, 1 µm. (**C**) Fluorescence intensity profile of TCs with two patches. For each 30 h TC (10 total from three biological replicates), a line was drawn through the apparent patch centers to the cell membrane at each end, as shown for a representative TC. The line length was normalized to 1. The fluorescence intensity of each channel was measured from 0 to 1 in increments of 0.04 along the line and normalized to its maximum, which was assigned a value of 1. Line, median. Shaded region, 95% credible interval. (**D**) Fluorescence intensity profile of spores with two patches. For each 42 h spore (10 total from three biological replicates), a line was drawn along the periphery starting at one patch where it was closest to the other, as shown for a representative spore. The line length was normalized to 1, and the fluorescence intensities were measured, normalized, and graphed as in panel C. (**E**) Enlarged image of representative spores stained with FM 4-64, but not DAPI, as a control. The image was acquired at 42 h in the red channel. Arrows point to arcs of intense membrane fluorescence. Bar, 1 µm.

We used the DNA-binding dye DAPI to visualize nucleoids in developing cells within NFBs. We found that DAPI blocked *M. xanthus* development, so we acquired images of different NFBs at different times PS. Optical sections near the base of NFBs showed mostly rods at 24 h, rods and TCs at 30 h, and mostly spores at 42 h (Fig. 2A), consistent with the results shown in Fig. 1A. Enlarged images of representative cells show that DNA occupied primarily the central portion of rods at 24 h (Fig. 2B; Fig. S6A), consistent with the localization of DNA during vegetative growth (21) (Fig. S6A), but the DNA appeared to be more condensed in developing rods, and indeed the nucleoid length was less as a proportion of cell length compared to vegetative cells (Fig. S6B). At 30 h, TCs exhibited DNA in one fluorescent patch (Fig. 2B, left panels), in a crescent along one side of the cell (center panels), or in two patches (right panels). At 42 h, spores showed DNA in one patch (Fig. 2B, left panels), in two patches (center panels), or distributed throughout the cytoplasm (right panels). Mature spores contain sufficient DNA to fill their cytoplasm as judged by DAPI staining (18), yet we observed localized DNA in some spores and in the majority of rods and TCs, likely due to DNA condensation. As noted above for TetR-YFP foci, not all the DNA is expected to be seen, owing to the thinness of the optical sections. Since entire chromosomes are much larger than the *tetO* arrays, we expected chromosomal DNA fluorescent patches to be larger than TetR-YFP foci, and this was the case. The larger patches are easier to detect than foci, but more likely to overlap. Finding one apparent patch in developing cells could reflect overlap or a lack of chromosome replication or segregation.

The two DAPI-stained DNA fluorescent patches observed in some TCs and spores appeared to be well-separated, as if two nucleoids had segregated. To quantify the separation, we examined the fluorescence intensity profiles. For TCs at 30 h PS, we drew a line through the apparent centers of the two patches and extending to the cell membrane at each end (Fig. 2C, left). The graph shows the normalized intensity of fluorescence from the DNA and membrane stains plotted versus the normalized distance along the line drawn, for 10 cells (Fig. 2C, right). On average, the DNA fluorescence intensity reached maxima of ~0.7 at distances of 0.3 and 0.7, indicating that the apparent centers of the two patches were separated by ~40% of the distance between the cell membrane at each end of the line. Importantly, the DNA fluorescence intensity reached ~0.5 at a distance of 0.5, midway between the maxima and similar in intensity to the minima of ~0.4 observed at each end of the line. We conclude that two chromosomal DNA fluorescent patches are well-separated in some TCs, providing evidence for nucleoid segregation during the shape change of developing cells.

To quantify the separation of DAPI-stained DNA fluorescent patches in spores at 42 h PS, a different approach was required because the two patches were at the periphery and there was little or no co-localized membrane staining. Therefore, we drew a line along the spore periphery, starting at one patch where it was closest to the other (Fig. 2D, left). On average, the DNA fluorescence intensity reached maxima of ~0.8 at distances of 0 and 0.6, and minima of ~0.4 between the maxima, indicating that the two patches were well-separated (Fig. 2D, right). Interestingly, membrane fluorescence intensity was out of phase with DNA fluorescence intensity. This apparent localization of membrane staining was not due to the DNA stain, since we observed localized FM 4-64 staining in the majority of spores at 42 h without DAPI addition (Fig. 2E). The out-of-phase nature of the DNA and membrane fluorescence intensities (Fig. 2D) suggests that DNA association with membrane interferes with FM 4-64 staining and/or that areas of intense FM 4-64 staining reflect a difference in the membrane that inhibits DNA association. We favor the latter hypothesis since spores with only one apparent DNA fluorescent patch typically exhibited two arcs of intense FM 4-64 staining that did not co-localize with the patch (Fig. 2B, left panels at 42 h). Careful examination of spores that appeared to have DNA distributed throughout the cytoplasm also typically revealed two arcs of intense FM 4-64 staining that did not co-localize with DNA staining (Fig. 2B, right panels at 42 h). In any case, some spores exhibited two well-separated chromosomal DNA fluorescent patches at their periphery, providing evidence for nucleoid segregation.

### mNeonGreen-FruA allows visualization of segregated nucleoids in a portion of TCs and spores without blocking development

**Fig 3:**
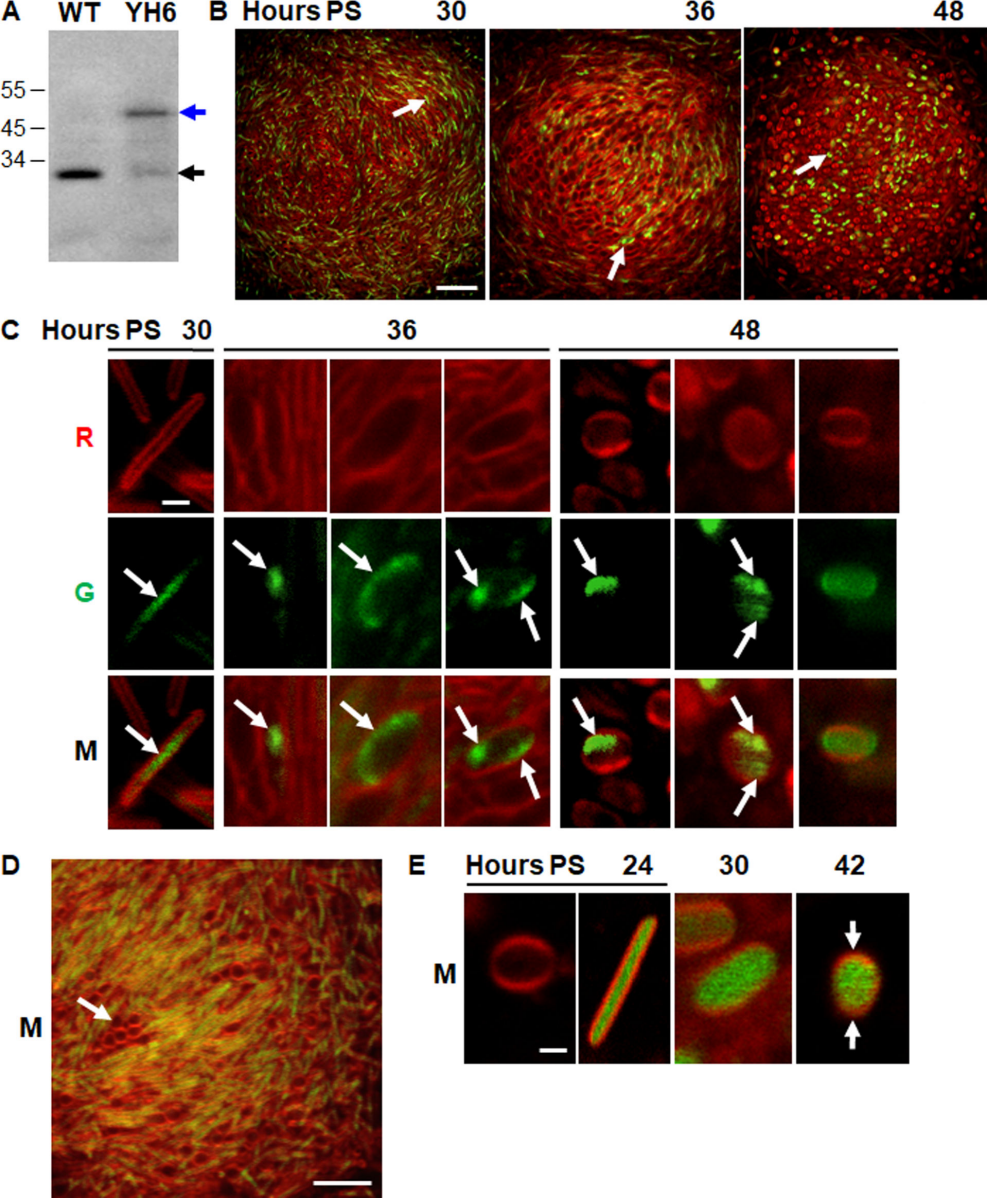
Segregated nucleoids can be observed in a portion of the TCs and spores without blocking development. *M. xanthus* strains were starved under submerged culture conditions. (**A**) Immunoblot analysis. Samples of wild-type (WT) strain DK1622 and strain YH6 were collected at 30 h PS, and equal volumes were analyzed by immunoblot with FruA antibodies. The blue arrow indicates a protein of the expected size for mNeonGreen-FruA, and the black arrow indicates the expected size for native FruA. The position of migration of molecular weight markers of the indicated size in kDa is shown on the left. (**B**) Visualization of nucleoids using mNeonGreen-FruA during development. FM 4-64 (5 µg/mL) was added at the start of starvation to a 1:3 mixture of YH6 and DK1622. Confocal images were acquired at the indicated times PS to show mNeonGreen-FruA fluorescence (green) and FM 4-64 staining of the cell membrane (red). Images show an optical section near the base of the same NFB over time, with the red and green channels merged. Arrows indicate a rod-shaped cell at 30 h, a TC at 36 h, and a spore at 48 h. Bar, 20 µm. (**C**) Enlarged images of representative cells. Images show rods at 30 h, TCs at 36 h, and spores at 48 h in the red (R), green (G), and merged (M) channels with one or two patches of green fluorescence (arrows). Bar, 1 µm. (**D**) Image of YH6 developed alone. The image shows an optical section near the base of a representative NFB at 30 h. Group of round cells lacking green fluorescence (arrow). Bar, 5 µm. See Fig. S7A for more images. (**E**) Enlarged images of representative cells engineered to produce mNeonGreen (not fused to FruA) as a control. Strain YH7 alone was treated with vanillate (0.5 mM) (in addition to FM 4-64) at the start of starvation. Images show a round cell and a rod at 24 h, TCs at 30 h, and a spore at 42 h. Arrows point to arcs of intense membrane fluorescence. Bar, 1 µm. See Fig. S7B for more images.

DAPI allowed nucleoid visualization but blocked development. For comparison using a different method, and to allow nucleoid visualization in developing cells of the same NFB over time, we engineered strain YH6, which produces mNeonGreen-FruA under control of the native *fruA* promoter at the native chromosomal locus. FruA is a developmental transcription factor that has been shown to bind cooperatively with MrpC to numerous DNA sites (23). We found that YH6 was delayed for development by ~6 h (Fig. S7A) compared to wild-type DK1622 (Fig. 2A). Immunoblot analysis with FruA antibodies detected a protein of the expected size (51 kDa) for mNeonGreen-FruA in YH6 extracts at 30 h PS, and a less abundant species matching native FruA in size (24.7 kDa) (Fig. 3A). The minor species may be a proteolytic fragment of mNeonGreen-FruA resulting from cleavage near the fusion junction. mNeonGreen-FruA in YH6 extracts was less abundant than FruA in wild-type DK1622 extracts, which may account for the delayed development of YH6. Alternatively or in addition, mNeonGreen-FruA may not be fully functional. In any case, YH6 develops and accumulates considerable mNeonGreen-FruA, so if the fusion protein binds to numerous DNA sites, it should allow nucleoid visualization.

To facilitate the potential observation of nucleoids in YH6, we mixed it with DK1622 at a cell ratio of approximately one to three (1:3). The 1:3 ratio provided separation between YH6 cells. Like YH6 alone (Fig. S7A), development of the 1:3 mixture was delayed by ~6 h (Fig. 3B) compared to DK1622 alone (Fig. 2A), so we imaged NFBs at times 6 h later PS than shown in Fig. 1 and 2. Optical sections near the base of the same NFB over time showed mostly rods at 30 h, rods and TCs at 36 h, and mostly spores at 48 h (Fig. 3B), consistent with the results shown in Fig. 1A and 2A, except reaching a similar stage of development 6 h later. Importantly, mNeonGreen-FruA appeared to allow nucleoid visualization (Fig. 3B) with similar localization as DAPI-staining of DNA (Fig. 2A). Enlarged images of representative cells show that mNeonGreen-FruA fluorescence occupied primarily the central portion of rods at 30 h (Fig. 3C). TCs at 36 h exhibited mNeonGreen-FruA fluorescence in one patch (Fig. 3C, left panels), in a crescent along one side of the cell (center panels), or in two patches (right panels). At 48 h, spores showed mNeonGreen-FruA fluorescence in one patch (Fig. 3C, left panels), in two patches (center panels), or distributed throughout the cytoplasm (right panels). The patterns of mNeonGreen-FruA fluorescence in YH6 cells are very similar to the patterns of DAPI fluorescence in DK1622 cells at a similar stage of development 6 h earlier (Fig. 2B). We conclude that both methods allow visualization of nucleoids, which are segregated in some TCs and spores, and mNeonGreen-FruA does so without blocking development.

Since Fig. 3B and C shows a 1:3 mixture of YH6:DK1622 cells, 75% of the cells did not express mNeonGreen-FruA, so we examined YH6 developed alone for the presence of round cells lacking cytoplasmic mNeonGreen-FruA fluorescence at 30 h, and we observed such cells (Fig. S7A; Fig. 3D). These results were consistent with the absence of TetR-YFP fluorescence in round YH5 cells at 24 h (Fig. 1D, leftmost panel; Fig. S2B, blue arrow) and in round YH3 and YH4 cells at 24 h (Fig. 1A; Fig. S2A). As noted already, these round cells present early in development may be spheroplasts destined for lysis. The putative spheroplasts appear to be unable to maintain the level or proper folding of fluorescent proteins.

As a control, we engineered strain YH7 to ectopically produce mNeonGreen (not fused to FruA) under the control of a vanillate-inducible promoter. The timing of YH7 development was normal (Fig. S7B), presumably because FruA is produced normally and mNeonGreen does not interfere with development. mNeonGreen fluorescence was distributed throughout the cytoplasm of YH7 rods, TCs, and spores (Fig. 3E), as expected, showing that localized mNeonGreen-FruA fluorescence in developing YH6 cells (Fig. 3C) depends on the FruA portion of the fusion protein. Round YH7 cells lacking cytoplasmic mNeonGreen fluorescence are present at 24 h (Fig. 3E, leftmost panel), consistent with the notion that these cells are spheroplasts unable to maintain the level or proper folding of fluorescent proteins and destined for lysis. Most spores of YH6 (Fig. 3C) and YH7 (Fig. 3E) showed two arcs of intense FM 4-64 fluorescence at opposite sides of their periphery, consistent with spores stained with FM 4-64 and DAPI (Fig. 2D) or with FM 4-64 alone (Fig. 2E).

### Fluorescence from mNeonGreen-FruA and DAPI co-localizes in cells

**Fig 4:**
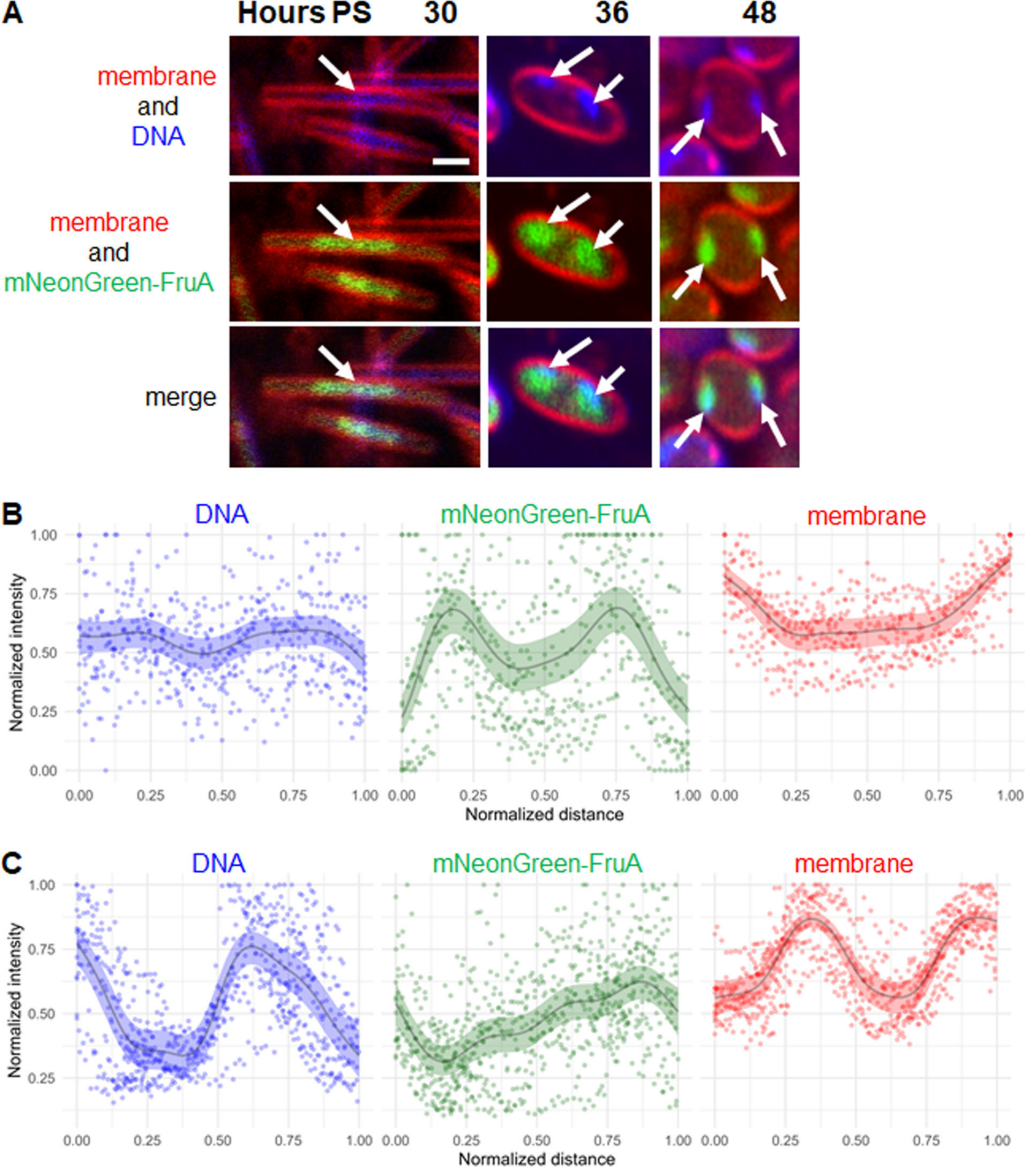
Co-localization of fluorescence from mNeonGreen-FruA and DAPI in developing cells. (**A**) Visualization of nucleoids. *M. xanthus* strain YH6 was starved under submerged culture conditions. FM 4-64 (5 µg/mL) was added at the start of starvation. DAPI (10 µg/mL) was added 30 min before imaging. Confocal images were acquired at the indicated times PS to show FM 4-64 staining of the cell membrane (red), DAPI staining of DNA (blue), and mNeonGreen-FruA fluorescence (green). Enlarged images of representative cells show rods at 30 h, TCs at 36 h, and spores at 48 h with one or two patches of fluorescence from DAPI and/or mNeonGreen-FruA (arrows). Bar, 1 µm. See Fig. S7C for more images. (**B**) Fluorescence intensity profile of TCs with two patches. Analysis of 36 h TCs (10 total from three biological replicates) was performed and graphed as described in Fig. 2C legend. (**C**) Fluorescence intensity profile of spores with two patches. Analysis of 48 h spores (10 total from three biological replicates) was performed and graphed as described in Fig. 2D legend.

To examine fluorescence from mNeonGreen-FruA, DAPI, and FM 4-64 in the same cells during development, we subjected YH6 alone to submerged culture development. Optical sections near the base of different NFBs showed mostly rods at 30 h PS, rods and TCs at 36 h, and mostly spores at 48 h (Fig. S7C), consistent with the results for an NFB formed by YH6 alone and imaged at different times without DAPI staining (Fig. S7A). Enlarged images of representative cells show that DAPI and mNeonGreen-FruA fluorescence overlapped primarily in the central portion of rods at 30 h. At 36 and 48 h, DAPI and mNeonGreen-FruA fluorescence overlapped in patterns consistent with those described for TCs and spores, respectively, when only mNeonGreen-FruA fluorescence was visualized in YH6 (Fig. 3C) or when only DAPI fluorescence was visualized in DK1622 at a similar stage of development 6 h earlier (Fig. 2B). The patches of mNeonGreen-FruA fluorescence typically appeared to be slightly larger than the patches of DAPI fluorescence (Fig. 4A), perhaps due to differences in detection sensitivity and/or DNA binding (e.g., DAPI binds preferentially to AT-rich regions of DNA [24], which are infrequent in the ~70% GC content *M. xanthus* genome [13, 25], whereas the GGGYRN_4-6_YGGG consensus sequence for FruA binding is GC-rich [26]). Figure 4A shows a TC at 36 h and a spore at 48 h in which two patches of DAPI and mNeonGreen-FruA fluorescence overlapped, strongly supporting the interpretation that both methods allow visualization of segregated nucleoids in developing cells.

To quantify the separation between nucleoids in YH6 TCs and spores, we analyzed the DAPI and mNeonGreen-FruA fluorescence intensity profiles as described for DAPI-stained DNA in DK1622 (Fig. 2C and D). For lines drawn through the apparent centers of two patches in TCs, the normalized DAPI and mNeonGreen-FruA fluorescence intensity profiles were similar, with the latter showing greater maxima and lesser minima on average (Fig. 4B). For lines drawn along the periphery of spores, starting at one patch where it was closest to the other, the normalized profiles were similar, but in this case, the DAPI signal showed greater maxima on average (Fig. 4C). Both signals were out of phase with the FM 4-64-stained membrane signal, consistent with the out-of-phase nature of the DAPI and FM 4-64 signals in DK1622 (Fig. 2D). We conclude that mNeonGreen-FruA and DAPI fluorescence co-localize in YH6, allowing quantification of nucleoid separation in TCs and spores using either signal and yielding similar results as DAPI in DK1622.

### Segregated nucleoids can be observed in ~40% of TCs and spores

**Fig 5:**
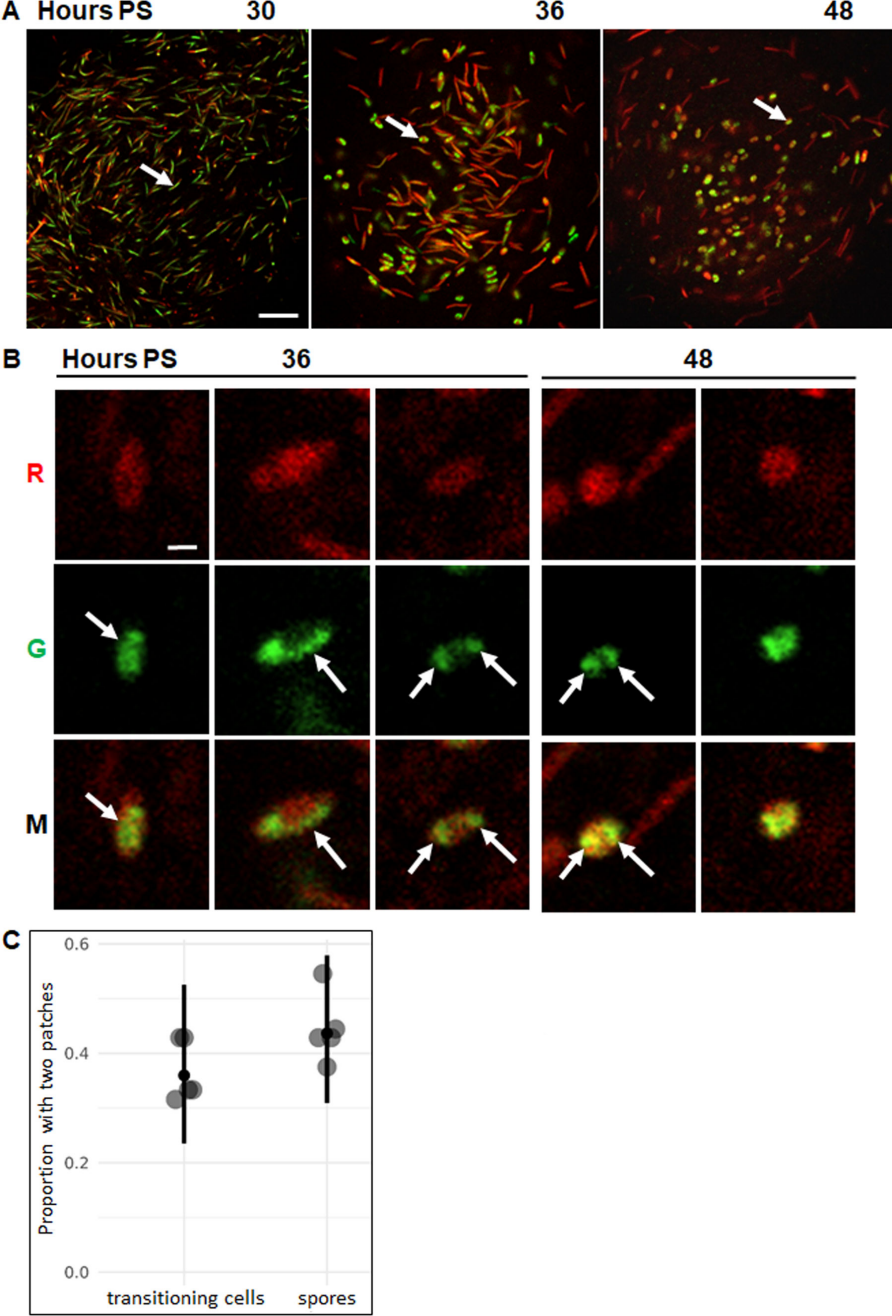
Observation of segregated nucleoids in ~40% of TCs and spores. (**A**) Visualization of cell shape and nucleoids during development. *M. xanthus* strain YH9 was mixed with wild-type strain DK1622 at a ratio of 1:3, and the mixture was subjected to starvation under submerged culture conditions. Vanillate (0.5 mM) was added at the start of starvation. Confocal images were acquired at the indicated times PS to show fluorescence of cytoplasmic tdTomato (red) and DNA-bound mNeonGreen-FruA (green) in YH9 cells. Images show an optical section near the base of the same NFB over time, with the red and green channels merged. Arrows indicate a rod-shaped cell at 30 h, a TC at 36 h, and a spore at 48 h. Bar, 20 µm. (**B**) Enlarged images of representative cells in maximum intensity projections of *z*-stacks. Images show TCs at 36 h and spores at 48 h in the red (R), green (G), and merged (M) channels with one or two patches of green fluorescence (arrows). These cells did not appear to overlap with any other cell in the *z*-stack projection. Bar, 1 µm. (**C**) Proportion of TCs and spores with segregated nucleoids. Cells that did not appear to overlap with any other cell in the *z*-stack projection were classified as having one or two patches of green fluorescence. The graph shows the proportion of TCs and spores with two patches from five NFBs (large dots) at 36 h (total of 109 TCs classified) and 48 h (total of 82 spores classified), the median (small dot), and the 95% credible interval (vertical line).

Because FM 4-64 stained the membrane of all the cells, which were packed inside NFBs in various orientations, it was challenging to determine the shape of some individual cells in a single optical section (0.5 µm). Furthermore, even though DAPI-stained or mNeonGreen-FruA-bound nucleoids were larger than TetR-YFP foci, nucleoids could be missed owing to the thinness of the optical section, so we wanted to collect *z*-stacks of optical sections to enhance nucleoid quantification. Therefore, we modified YH6, which produces mNeonGreen-FruA under control of the native *fruA* promoter as we have already described, to produce tdTomato ectopically under the control of a vanillate-inducible promoter, resulting in strain YH9. We reasoned that red fluorescence from tdTomato would be distributed throughout the cytoplasm of cells and allow their shape to be determined in most cases, while mNeonGreen-FruA would allow visualization of most nucleoids in the same cells. We found that YH9 was delayed for development by ~6 h (Fig. S7D) compared to DK1622 (Fig. 2A), as expected since YH6 was similarly delayed (Fig. S7A). We mixed YH9 and DK1622 cells at a 1:3 ratio to provide separation between YH9 cells, and development was delayed by ~6 h (Fig. 5A), as seen for the 1:3 mixture of YH6 and DK1622 (Fig. 3B), so we imaged NFBs at the same times PS. Optical sections near the base of the same NFB over time showed mostly rods at 30 h, rods and TCs at 36 h, and mostly spores at 48 h (Fig. 5A). At 36 and 48 h, we collected *z*-stacks of optical sections from near the base of NFBs to 5 µm up. Resolution decreased in optical sections farther than 5 µm up.

We projected each *z*-stack onto a plane orthogonal to the *z*-axis to create a maximum intensity projection. Only the voxels with maximum intensity along the *z*-axis were projected onto the plane, resulting in a partial representation of the *z*-stack in two dimensions. We ignored cells that overlapped with other cells. Among the remaining cells, enlarged images of representative cells show that TCs at 36 h PS exhibited mNeonGreen-FruA fluorescence in one patch (Fig. 5B, left panels), in a crescent along one side of the cell (center panels), or in two patches (right panels), consistent with the results observed for YH6 (Fig. 3C). At 48 h, spores showed mNeonGreen-FruA fluorescence in two patches (Fig. 5B, left panels) or distributed throughout the cytoplasm (right panels). We did not observe spores with mNeonGreen-FruA fluorescence in one patch, in contrast to the results for YH6 (Fig. 3C, left panels at 48 h). However, we examined only one optical section of YH6, so we might have missed seeing a second patch, owing to the thinness of the optical section. Conceivably, a second patch could be missed in a maximum intensity projection of a *z*-stack, but this should occur infrequently if segregated nucleoids are well-separated, as our data show (Fig. 2 and 4). Therefore, we determined the proportions of TCs and spores with two patches of mNeonGreen-FruA fluorescence in the maximum intensity projections of five NFBs at 36 and 48 h, respectively. For both TCs and spores, ~40% of the cells exhibited two patches, and the means were within the uncertainty of our data (Fig. 5C). We conclude that segregated nucleoids can be observed in ~40% of TCs and spores. Most TCs had a crescent-shaped nucleoid along one side, and most spores had mNeonGreen-FruA fluorescence distributed throughout the cytoplasm.

### Segregated nucleoids can be observed in ~10%–20% of TCs and spores during glycerol-induced sporulation

**Fig 6:**
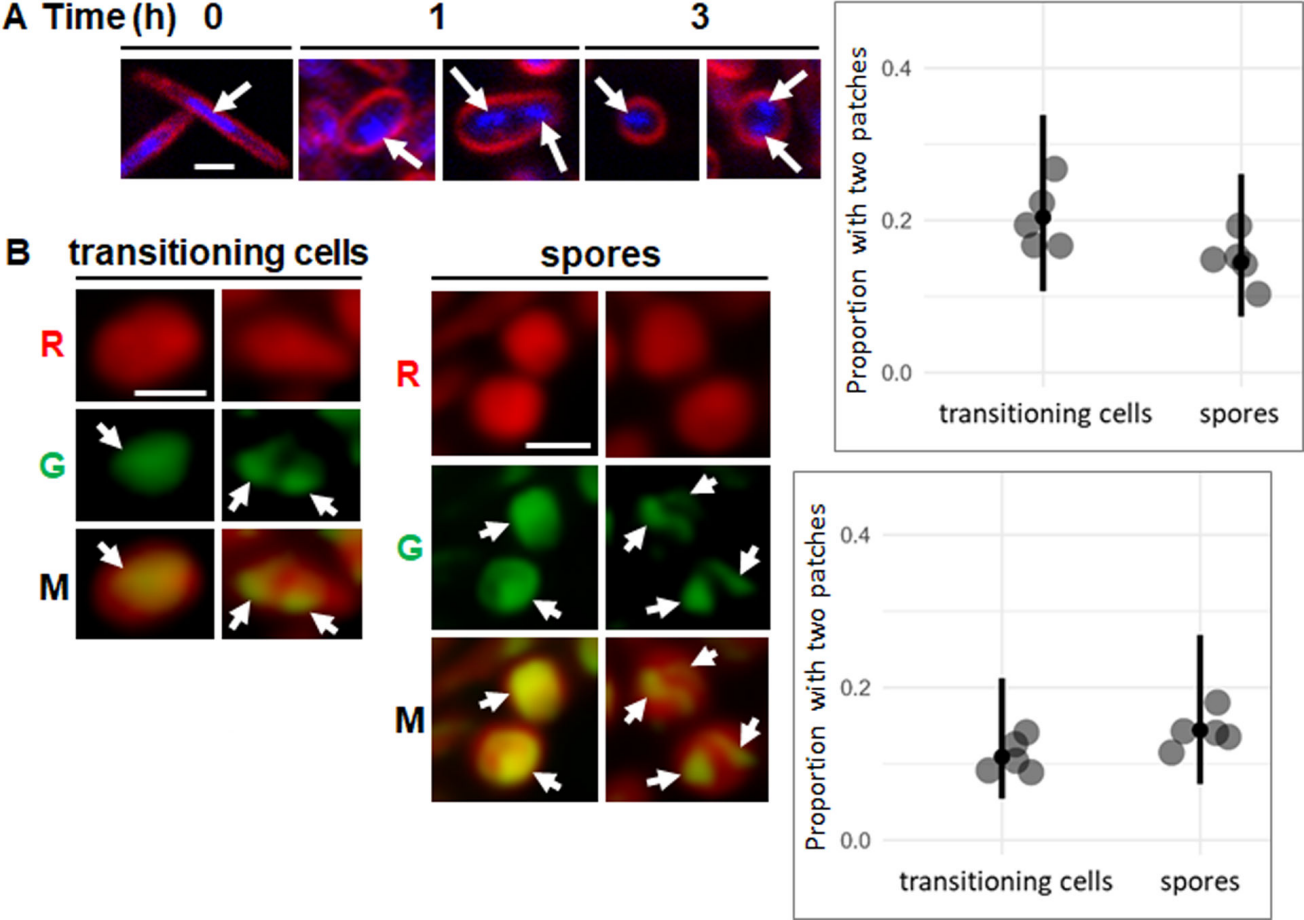
Observation of segregated nucleoids in ~10%–20% of TCs and spores during glycerol-induced sporulation. (**A**) Visualization of DAPI-stained DNA. To *M. xanthus* strain DK1622 growing in nutrient medium, FM 4-64 (5 µg/mL) was added. Samples were taken before the addition of glycerol (0.5 M) (designated 0 h) and at 1 and 3 h post-addition. DAPI (10 µg/mL) was added 30 min before imaging. Confocal images were acquired to show DAPI staining of DNA (blue) and FM 4-64 staining of the cell membrane (red). Enlarged images of representative cells in maximum intensity projections of *z*-stacks. Images show rods at 0 h, TCs at 1 h, and spores at 3 h with one or two DAPI-stained DNA fluorescent patches (arrows). Bar, 1 µm. The graph shows the proportion of TCs and spores with two patches from five biological replicates (large dots) at 1 h (total of 147 TCs classified) and 3 h (total of 136 spores classified), the median (small dot), and the 95% credible interval (vertical line). (**B**) Visualization of cell shape and nucleoids. To *M. xanthus* strain YH9 growing in nutrient medium with vanillate (0.5 mM), glycerol (0.5 M) was added, and a sample was taken 3 h later. Confocal images were acquired to show fluorescence of cytoplasmic tdTomato (red) and DNA-bound mNeonGreen-FruA (green). Enlarged images of representative cells in maximum intensity projections of *z*-stacks show red (R), green (G), and merged (M) channels with one or two patches of green fluorescence (arrows). Bar, 1 µm. The graph shows the proportion of TCs (total of 108 classified) and spores (total of 125 classified) with two patches from five biological replicates (large dots), the median (small dot), and the 95% credible interval (vertical line).

*M. xanthus* can form spores not only in fruiting bodies during starvation, but also without starvation or fruiting body formation, when growing cells are treated with certain chemicals, including glycerol (27). A previous study of glycerol-induced spores showed that their chromosome copy number varies from one to two or even more (18). To examine nucleoid localization during the unicellular process of glycerol-induced sporulation, we stained DK1622 with DAPI and FM 4-64 to visualize DNA and the cell membrane, respectively. Since we visualized cells in a monolayer between an agarose pad and a glass cover slip (rather than packed inside NFBs), we collected *z*-stacks of optical sections from just below cells in the monolayer to 5 µm up (i.e., well above the cells) and created maximum intensity projections as already described. As expected (21) (Fig. S6A), the DNA occupied primarily the central portion of growing rods (Fig. 6A, 0 h). We observed mostly TCs and spores at 1 and 3 h after glycerol addition, respectively, and both exhibited DAPI-stained DNA in one patch (Fig. 6A, left panels) or in two patches (right panels). We did not observe any cells with more than two patches. In contrast to our observations of DAPI-stained DNA in TCs and spores within NFBs (Fig. 2B), we did not observe glycerol-induced TCs containing a crescent-shaped nucleoid along one side or spores with decondensed nucleoids. We quantified the proportion of TCs and spores at 1 and 3 h, respectively, with two DAPI-stained DNA patches, which were ~20% and ~15%, respectively, but both means were within the uncertainty of our data (Fig. 6A graph). We conclude that segregated nucleoids can be observed in ~15%–20% of TCs and spores with DAPI staining of DNA during glycerol-induced sporulation.

We found that YH9 grown in the presence of vanillate to induce tdTomato expressed mNeonGreen-FruA by 3 h after glycerol addition, so we visualized cells and collected *z*-stacks at that time, then created maximum intensity projections as already described. Red fluorescence from tdTomato revealed both TCs and spores, and enlarged images of representative cells show that both exhibited mNeonGreen-FruA fluorescence in one patch (Fig. 6B, left panels) or in two patches (right panels). We did not observe any cells with more than two patches. In agreement with our observations of DAPI-stained DNA in glycerol-induced TCs and spores (Fig. 6A), we did not observe TCs containing a crescent-shaped nucleoid along one side or spores with fully decondensed nucleoids. However, we note that nucleoids appear less condensed in YH9 expressing mNeonGreen-FruA at ~3 h after glycerol addition (Fig. 6B) than in DK1622 stained with DAPI at 1 or 3 h after adding glycerol (Fig. 6A). One possible explanation is that DAPI addition to DK1622 arrested a decondensation process that continued in YH9 for ~15–30 min during sample preparation and *z-*stack collection. We quantified the proportion of TCs and spores with two patches of mNeonGreen-FruA fluorescence, which were ~10% and ~15%, respectively, but both means were within the uncertainty of our data (Fig. 6B graph). These results are similar to those with DAPI staining of DNA (Fig. 6A), supporting our conclusion that segregated nucleoids can be observed in ~10%–20% of TCs and spores during glycerol-induced sporulation.

## DISCUSSION

Our results show that sister chromosomes often segregate when rod-shaped cells transition to round spores during both starvation- and glycerol-induced development of *M. xanthus*. Staining cells with the DNA-binding dye DAPI or producing the DNA-binding transcription factor mNeonGreen-FruA showed two well-separated fluorescent patches in ~40% of the TCs and spores in starvation-induced NFBs. Most of the other TCs had a crescent-shaped nucleoid along one side that may be indicative of ongoing segregation. The nucleoids appeared to have decondensed in the majority of spores. Only ~10%–20% of TCs and spores exhibited segregated nucleoids during the rapid process of unicellular glycerol-induced sporulation. We discuss the potential mechanism of developmental nucleoid segregation and possible reasons for it. We also discuss the intriguing possibility that the subpopulation of round cells lacking cytoplasmic fluorescence, which we observed in several experiments early in development, may identify spheroplasts at a late stage of a dying process that ends with lysis.

### The potential mechanism of chromosome segregation during cell shape change

Understanding the mechanism of nucleoid segregation during development could allow its manipulation and the determination of its importance for developmental progression. *M. xanthus* uses a *parABS* system for chromosome segregation in growing cells (21, 28). ParB binds to *parS* sequences in the chromosome. Based on a large amount of work on *parABS* systems that partition bacterial chromosomes or plasmids (29), the ParB/*parS* complexes bind to ParA-ATP dimers bound nonspecifically to nucleoid DNA, stimulating ParA ATPase activity and releasing ParA-ADP from both the nucleoid and ParB/*parS*. This is proposed to create gradients of nucleoid-bound ParA-ATP dimers that move ParB/*parS* complexes toward the cell poles via cycles of diffusion, capture, and release (30–33). It is likely that chromosome segregation during *M. xanthus* development is mediated by its *parABS* system. To test this hypothesis, conditional mutant strains are necessary because ParA and ParB are essential for cell viability (21, 28). A strain engineered to produce ParB under the control of a vanillate-inducible promoter has been described (28), which could be subjected to vanillate withdrawal at the onset of development or closer to the time that rods begin transitioning to spores, depending on the kinetics of ParB depletion. This may allow manipulation of nucleoid segregation and determination of its effects on the formation of TCs and spores.

Several other multiprotein systems with important roles in chromosome segregation during growth have been identified recently. The BacNOP/PadC scaffold helps position ParB/*parS* complexes and ParA (34) and has a different yet redundant role with the structural maintenance of chromosome complex (35). Our study raises questions about possible functions of these multiprotein systems in nucleoid segregation during developmental cell shape change. The *M. xanthus* genome also encodes three ParA-like proteins (18), but to our knowledge, their potential functions in chromosome segregation during growth or development have not been investigated.

Some of our results suggest the mechanism of sister chromosome segregation may differ in developing and growing cells. We did not detect a biased chromosome arrangement in developing rods, TCs, or spores (Fig. 1C), whereas predivisional cells during growth show a highly biased *ori-ter-ter-ori* arrangement of sister chromosomes (21) (Fig. S1C). Predivisional cells are ~6-8 µm long, and *tetO* arrays located at 33° or 270° from *ori* lead to TetR-YFP foci ~3–4 µm apart (21). Developing rods are ~6 µm long (Fig. S3) and *tetO* arrays at the same locations lead to TetR-YFP foci ~0.75 µm apart (Fig. 1C). The TetR-YFP foci would be ~3 µm apart if sister chromosomes were arranged *ori-ter-ter-ori*, as in predivisional cells. The >2 µm discrepancy supports a different chromosome arrangement in developing rods (e.g., *ori-ori-ter-ter*, Fig. S1C) than in predivisional cells. We cannot rule out the possibility that a difference in chromosome condensation contributes to the discrepancy, but developing rods and vegetative cells differed in nucleoid length by only ~0.5–1.0 µm (Fig. S6B).

Interestingly, TCs showed neither a biased chromosome arrangement nor a significant difference in the distance between TetR-YFP foci compared to developing rods (Fig. 1C). The latter observation is surprising since ~40% of TCs had segregated nucleoids (Fig. 5C) and most other starvation-induced TCs had a crescent-shaped nucleoid along one side (Fig. 2B, 3C and 5B). Perhaps the two chromosomal loci we examined are not segregated as much as other parts of the nucleoids that are visible by DAPI staining and mNeonGreen-FruA binding. Testing *tetO*-arrays located elsewhere in the chromosome (e.g., near *ter* at 192° from *ori*) and ParB-YFP binding to *parS* near *ori* (21), and combining these approaches with DAPI staining could help resolve remaining questions about the arrangement of sister chromosomes in TCs.

The crescent-shaped nucleoid along one side of starvation-induced TCs, but not glycerol-induced TCs, is intriguing. A recent report highlighted the importance of transertion (coupled transcription, translation, and insertion of membrane proteins) in nucleoid localization close to the inner membrane of rapidly growing *E. coli* (36). Transertion may contribute to the localization of the crescent-shaped nucleoid in starvation-induced *M. xanthus* TCs. In particular, synthesis of enzymes involved in peptidoglycan remodeling (37, 38), which accompanies ongoing shape change in TCs, could promote nucleoid localization to the inner membrane. The lack of a crescent-shaped nucleoid in glycerol-induced TCs suggests that other factors govern nucleoid shape. For example, the rapid cell shape change induced by glycerol may cause nucleoid expansion due to loss of confinement and altered molecular crowding (36, 39–41).

Spores are only ~2 µm in diameter (Fig. S3), so a biased chromosome arrangement may not have been detectable in our experiment. Tzeng and Singer (18) reported that both *ori* and *ter* regions of sister chromosomes localize to the periphery of mature (5-day-old) spores, commonly ~180° apart, but the *sdeK* locus localizes to the spore interior. In our experiment, *tetO-*arrays located at 33° or 270° from *ori* often localized to the periphery of spores at 42 h PS, but not 2 µm apart; rather, ~0.75 µm apart on average, similar to developing rods and TCs (Fig. 1C).

Whether the distance between sister chromosomal loci changes as rods transition to spores, and other important questions about the mechanism of chromosome segregation during cell shape change, can best be addressed by tracking chromosome dynamics in individual cells. However, it was challenging to determine the fate of TCs by making time-lapse movies of starvation-induced NFBs (17). Although TCs are not motile, many left the focal plane during 12 h of imaging, possibly due to lysis or movement (driven by shape change and/or movement of other TCs or rods). The rapid, unicellular process of glycerol-induced sporulation may be much more amenable to tracking of chromosome dynamics in developing cells. The two developmental processes share many features (27, 42, 43). One difference is that the chromosome copy number is more variable for glycerol-induced spores, with nearly equal proportions having one or two copies (18). Weaker coupling between DNA replication and cell shape change after glycerol addition may explain the approximately twofold lower proportion of TCs and spores with segregated nucleoids we observed (Fig. 6) compared to starvation-induced development (Fig. 5C). For cells with two copies of the chromosome during glycerol-induced sporulation, it remains to be seen whether segregation of sister chromosomes is as efficient and employs a similar mechanism as during starvation-induced development.

### Nucleoid segregation as a bet-hedging strategy or an evolutionary remnant

A compartmentalization event typically accompanies chromosome segregation. During bacterial growth, chromosome condensation and segregation precede cell division, so that progeny cells each receive a copy of the chromosome (12). During *B. subtilis* endosporulation, chromosome translocation across an asymmetrically positioned division septum distributes one copy of the chromosome to the forespore, while the other copy remains in the mother cell (44). However, we found that nucleoid segregation occurs without compartmentalization during *M. xanthus* development (Fig. 2 to 6). One possible explanation is that nucleoid segregation is a bet-hedging strategy to facilitate rapid cell division after spore germination, if nutrients become available prior to spore maturation. According to this hypothesis, newly formed spores with segregated nucleoids would be poised for cell division after germination, gaining a growth advantage in environments that often fluctuate in nutrient supply. On the other hand, since nucleoid segregation requires resources, perhaps it does not occur in some TCs and spores, enhancing spore survival in environments prone to prolonged starvation, and hence, hedging bets. In previous studies, nutrient addition to starving cells provided evidence for commitment to spore formation at about the time that cell shape change begins (45, 46). One of these studies suggested that some newly formed spores germinate in response to nutrients (45). Determining whether newly formed spores with segregated vs. non-segregated nucleoids differ in the pace of cell division after spore germination will require tracking of both chromosome dynamics and subsequent division of individual cells. Comparison of newly formed vs. mature spores is currently more tractable. Our results suggest that as spores mature, their nucleoids decondense. The nucleoids appeared to have decondensed in the majority of spores by 48 h PS (Fig. 5), and nucleoid localization was not observed by 72 h (Fig. S8). Perhaps the quiescent state of spores makes it difficult to maintain nucleoid condensation and segregation. Whether mature spores and newly formed spores differ in terms of how soon cell division occurs after germination can be investigated. This and many other questions would need to be answered to determine whether the nucleoid phenotypic heterogeneity we have observed fits the formal definition of bet hedging (47–50). For example, if newly formed spores with segregated nucleoids undergo cell division faster after germination than newly formed spores with non-segregated nucleoids, it would need to be shown that the latter are better adapted under some other condition, such as prolonged starvation.

Alternatively, nucleoid segregation during *M. xanthus* development could be an evolutionary remnant of ancestral events that included cell division to produce spores with one copy of the chromosome. To our knowledge, the chromosome copy number of myxobacterial spores other than those of *M. xanthus* has not been determined. Systematic determination of spore chromosome copy number throughout the myxobacterial phylogenetic tree, which is extensive (51), could test the evolutionary remnant hypothesis. The driving force for the evolution of spores with two copies of the chromosome might have been increased survival and the ability to germinate in the soil environment (18), resulting in loss of ancestral cell division.

### Round cells present early in development may be spheroplasts destined for lysis

Several of our experiments revealed a subpopulation of round cells that were distinct from TCs and spores. Under our submerged culture conditions of development, mounds form at ~18 h PS, and by this time, the number of rods remaining is only ~30% of the initial number (i.e., 70% have undergone lysis) (45, 52). Although many cells undergo lysis, they may not persist as round cells for long (see below). The round cells are unlikely to be spore precursors, since sonication-resistant spores are not detected until 27 h (52), yet we have observed round cells at 18 h (data not shown). Moreover, we have shown that TCs are elliptical (17), not round, and that both TCs and spores differ from the subpopulation of round cells in terms of their ability to exhibit fluorescence from TetR-YFP (Fig. 1D) and mNeonGreen-FruA (Fig. 3). The lack of fluorescence from the round cell subpopulation suggests they are unable to maintain the level or proper folding of fluorescent proteins. Round cells also did not exhibit DNA localization, as did the majority of rods and TCs, and many spores (Fig. 2). The lack of DNA localization in round cells suggests an inability to maintain DNA condensation and/or integrity. Altogether, the characteristics of round cells present early in development suggest they are spheroplasts destined for lysis.

The subpopulation of round cells we observed is smaller than the proportions of “dead” cells reported in prior studies of developing *M. xanthus* (53, 54). The previous studies used the LIVE/DEAD BacLight epifluorescence staining method (55) and showed that ~50% of the cells present at 24 h PS (53) or 18 h (54) stained with propidium iodide (red). These cells are often classified as “dead,” but are more accurately described as having a compromised cell envelope and therefore potentially dying or dead (53, 56). In any case, we observed <50% round cells at 24 h (Fig. S2B; Fig. 2A; Fig. S7B) or 30 h (Fig. 3D; Fig. S7A), so taken together with the previous studies (53, 54), our findings suggest that round cells are short-lived compared to developing cells with a compromised cell envelope. We propose that propidium iodide staining detects rods and perhaps TCs at an early stage of the dying process, and that round cells are spheroplasts at a later stage of the dying process, soon to undergo lysis. This could be examined by comparing propidium iodide staining with fluorescence from any of several strains described herein (YH5, YH6, or YH7) that allow identification of round cells. Our hypothesis predicts that more cells earlier in development would stain with propidium iodide than would in parallel experiments exhibit fluorescence indicative of round cells (i.e., round red fluorescence due to FM 4-64 membrane staining and no cytoplasmic fluorescence).

In conclusion, we discovered that nucleoids often segregate in TCs during *M. xanthus* development, raising numerous questions about the potential mechanism and why it occurs. We also found a subpopulation of cells early in development that may be spheroplasts destined for lysis, the fate of most cells during fruiting body formation (14, 15).

## MATERIALS AND METHODS

### Bacterial strains, plasmids, and primers

*M. xanthus* strains, plasmids, and primers used in this study are listed in Table S1. pYH3, which was used to induce production of TetR-YFP with IPTG, was constructed by replacing the *lacZ* gene of pMR3487 with a *tetR-yfp* fragment amplified from pLAU53 using tetR-YFP-F and tetR-YFP-R primers. pMR3487 was digested with XbaI and KpnI restriction enzymes. A Gibson assembly reaction was used to enzymatically join the overlapping DNA fragments (57). The reaction mixture was transformed into *E. coli* strain DH5α with outgrowth in Luria-Bertani (LB) liquid medium prior to plating on LB agar (1.5%) supplemented with 15 µg/mL tetracycline for selection at 37°C. The construct was verified by sequencing with pMR3487 F and pMR3487 R primers. pYH3 was electroporated (58) into *M. xanthus* strains SA4212, SA4118, and DK1622, resulting in strains YH3, YH4, and YH5, respectively.

pYH6 was used to replace *fruA* with *mNeonGreen-fruA* in the *M. xanthus* chromosome. To construct pYH6, DNA fragments corresponding to regions flanking the *fruA* start codon were amplified from *M. xanthus* chromosomal DNA using five flank FOR and five flank REV primers, and using three flank FOR and three flank REV primers. An *mNeonGreen* fragment was amplified from pFM13 (Penelope Higgs, personal communication) using GFP FOR and GFP REV primers. The three fragments were mixed with SmaI-digested pBJ114, and the fragments were enzymatically joined in a Gibson assembly reaction (57). The reaction mixture was transformed into *E. coli* strain DH5α, with outgrowth in LB liquid medium prior to plating on LB agar supplemented with 50 µg/mL kanamycin for selection at 37°C. The DNA sequence of the joined fragments was verified, and pYH6 was electroporated (58) into *M. xanthus* strain DK1622, with outgrowth in Casitone-Tris (CTT) liquid medium (1% Casitone, 10 mM Tris-HCl [pH 8.0], 1 mM KH_2_PO_4_-K_2_HPO_4_, 8 mM MgSO_4_, [final pH 7.6]) prior to plating on CTT agar (1.5%) supplemented with 40 µg/mL kanamycin for selection at 32°C. The positive-negative screening was carried out as described previously (59) with 40 µg/mL kanamycin and 5% galactose. Colonies that grew in the presence of galactose, but not in the presence of kanamycin, were tested by colony PCR using GFP FOR and GFP REV primers, and a strain that produced the expected PCR product was named YH6.

### Growth and development

*M. xanthus* was grown at 32°C on CTT agar or in CTTYE liquid medium (CTT with 0.2% yeast extract), shaking at 350 rpm. Starvation-induced development was performed under submerged culture conditions in 8-well μ-slides (Ibidi) with starvation buffer MC7 (10 mM morpholinepropanesulfonic acid [MOPS, pH 7.0], 1 mM CaCl_2_). Cells from log-phase cultures in CTTYE liquid were resuspended in MC7 at 1,000 Klett units as described previously (45). Cell suspension (26 µL) was added to MC7 (174 µL) in each well. Upon incubation at 32°C, cells formed a biofilm at the bottom of the well and underwent development. To visualize TetR-YFP foci in vegetative cells, cultures of YH4 were induced with IPTG during 4 h of exponential growth, then cells were sedimented (7,000 × *g*, 1 min) and resuspended at 1,000 Klett units in TPM buffer (10 mM Tris·HCl [pH 7.5], 1 mM KH_2_PO_4_·K_2_HPO_4_, 8 mM MgSO_4_) containing IPTG and FM 4-64. Glycerol-induced sporulation was performed by adding glycerol (0.5 M) to *M. xanthus* growing exponentially at 32°C in CTTYE liquid medium, shaking at 350 rpm.

### Microscopy

Images of NFBs were acquired with a Nikon A1 Laser Scanning Confocal Microscope configured on a Nikon Ti inverted platform with an XY automated stage and a 100× objective. Fluorescence from FM 4-64 and tdTomato was examined using a 560 nm laser for excitation and a 595/50 band pass emission filter. DAPI fluorescence was examined using a 402 nm laser for excitation and a 450/50 band pass emission filter. Fluorescence from mNeonGreen was examined using a 488 nm laser for excitation and a 525/50 band pass emission filter. Images “near the base of NFBs” were the first optical section above the bottom of the well in which cells could be clearly visualized.

### Image analysis

The distance between TetR-YFP foci and the dimensions of cells based on FM 4-64 fluorescence were measured using ImageJ software (60). Fluorescence intensity profiles were measured using the software of the Nikon A1 Laser Scanning Confocal Microscope. First, FM 4-64 fluorescence was used to visualize the cell membrane, then DAPI and/or mNeonGreen-FruA fluorescence was used to visualize nucleoids in cells selected for analysis. For each cell, a line was drawn across the cell (TCs) or along the periphery (spores), and the fluorescence intensities were measured and analyzed as described in the text and the figure legends. Maximum intensity projections were generated using ImageJ software (60) by applying the “Maximum Intensity” projection function (Image >Stacks > Z Project) to the *z*-stacks, with all slices included in the projection.

## SUPPLEMENTAL MATERIAL

Supplemental material jb.00328-25-s0001.pdf Table S1, Figure S1 to S8, and references.

ASM does not own the copyrights to Supplemental Material that may be linked to, or accessed through, an article. The authors have granted ASM a non-exclusive, world-wide license to publish the Supplemental Material files. Please contact the corresponding author directly for reuse.
