# Supplementary material for: Segregation of sister chromosomes during the shape change of developing Myxococcus xanthus cells
Source: J Bacteriol. 2025 Sep 26;207(10):e00328-25. doi: 10.1128/jb.00328-25 (PMC12548468; doi:10.1128/jb.00328-25)
Supplement: Supplemental material — Table S1, Figure S1 to S8, and references. [file jb.00328-25-s0001.pdf]

SUPPLEMENTAL MATERIAL

Segregation of sister chromosomes during the cellular shape change of developing *Myxococcus xanthus*

Y Hoang[1, 2], Yann S. Dufour[2], and Lee Kroos[1, 2 #]

[1]Department of Biochemistry and Molecular Biology, Michigan State University, East Lansing, Michigan, USA

[2]Department of Microbiology and Molecular Genetics, Michigan State University, East Lansing, Michigan, USA

Short title: Nucleoids segregate during *Mycococcus* development

[#]Address correspondence to Lee Kroos, kroos@msu.edu

Table S1 Bacterial strains, plasmids, and primers used in this study

Strain, plasmid, or primer	Description	Source or reference
**Strain**		
E. coli		
DH5α	λ^- ϕ80dlacZΔM15 Δ(*lacZYA-argF*)*U169 recA1 endA1 hsdR17*(r_K^- m_K^-) *supE44 thi-1 gyrA relA1*	[1]
M. xanthus		
DK1622	Laboratory wild-type strain	[2]
SA4212	*tetO*-array in mxan0733 region (pNV3)	[3]
SA4118	*tetO*-array in mxan5499 region (pMAT18)	[3]
YH3	P_{IPTG}-*tetR-yfp* in SA4212 using pYH3	This study
YH4	P_{IPTG}-*tetR-yfp* in SA4118 using pYH3	This study
YH5	P_{IPTG}-*tetR-yfp* in DK1622 using pYH3	This study
YH6	P_{fruA}-*mNeonGreen-FruA* in DK1622 using pYH6	This study
YH7	P_{van}-*mNeonGreen* in DK1622 using pYH7	[4]
YH9	P_{van}-*tdTomato* in YH6 using pYH9	This study
**Plasmid**		
pMR3487	1.38-kb-P_{IPTG}-MCS_A-P_{R4}::*lacI*, Tc^r	[5]
pLAU53	P_{ara}-*tetR-yfp*	[6]
pYH3	1.38-kb-P_{IPTG}-*tetR-yfp*_A-P_{R4}::*lacI*, Tc^r	This study
pBJ114	Backbone for gene replacements; *galK* Km^r	[7]
pYH6	P_{fruA}-*mNeonGreen-FruA*, Km^r	This study
pYH7	MXAN_0018-MXAN_0019-P_{R3-4}::*vanR*-P_{van}::*mNeonGreen*, Tc^r	[4]
pYH9	MXAN_0018-MXAN_0019-P_{R3-4}::*vanR*-P_{van}::*tdTomato*, Tc^r	[4]
**Primer**		
tetR-YFP-F	GGAATCTAGAATGGTGTCTAGATTAGATAAAAG	This study
tetR-YFP-R	GCCAGGTACCTTACTTGTACAGCTCGTC	This study
pMR3487 F	GTAAATGTGAGCACTCACAAT	This study
pMR3487 R	CCCGCACTCAGCTTGGAGGTG	This study
5 flank FOR	CGAATTCGAGCTCGGTACCCGTCGGCAGCATACACG TCTG	This study
5 flank REV	TCTTCACCTTTGCTGACCATGCGAAGGCCCCCCAGCC GT	This study
3 flank FOR	GCGGGCTCCGCGGCGGGCTCCGGCGAGTTCATGGC AACCAATCAAGCAGC	This study
3 flank REV	GGTCGACTCTAGAGGATCCCCCAATCTTCAGGTTGTC CGCG	This study
GFP FOR	ATGGTCAGCAAAGGTGAAGAAGAC	This study
GFP REV	GCCGGAGCCCGCCGCGGAGCCCGCGGAGCCCTTGT ACAGTTCGTCCATACCCATC	This study
Van-tdTom F	GATGCGAGGAAACGCATATGGTGAGCAAGGGCGAG	[4]

Van-tdTom R	GTACGCGTAACGTTCGAATTCTTACTTGTACAGCTCGTCCATG	[4]
pMR3691 MCS G-F	CACGATGCGAGGAAACGCA	[4]
pMR3691 MCS G-R	CACCGGTACGCGTAACGTTC	[4]

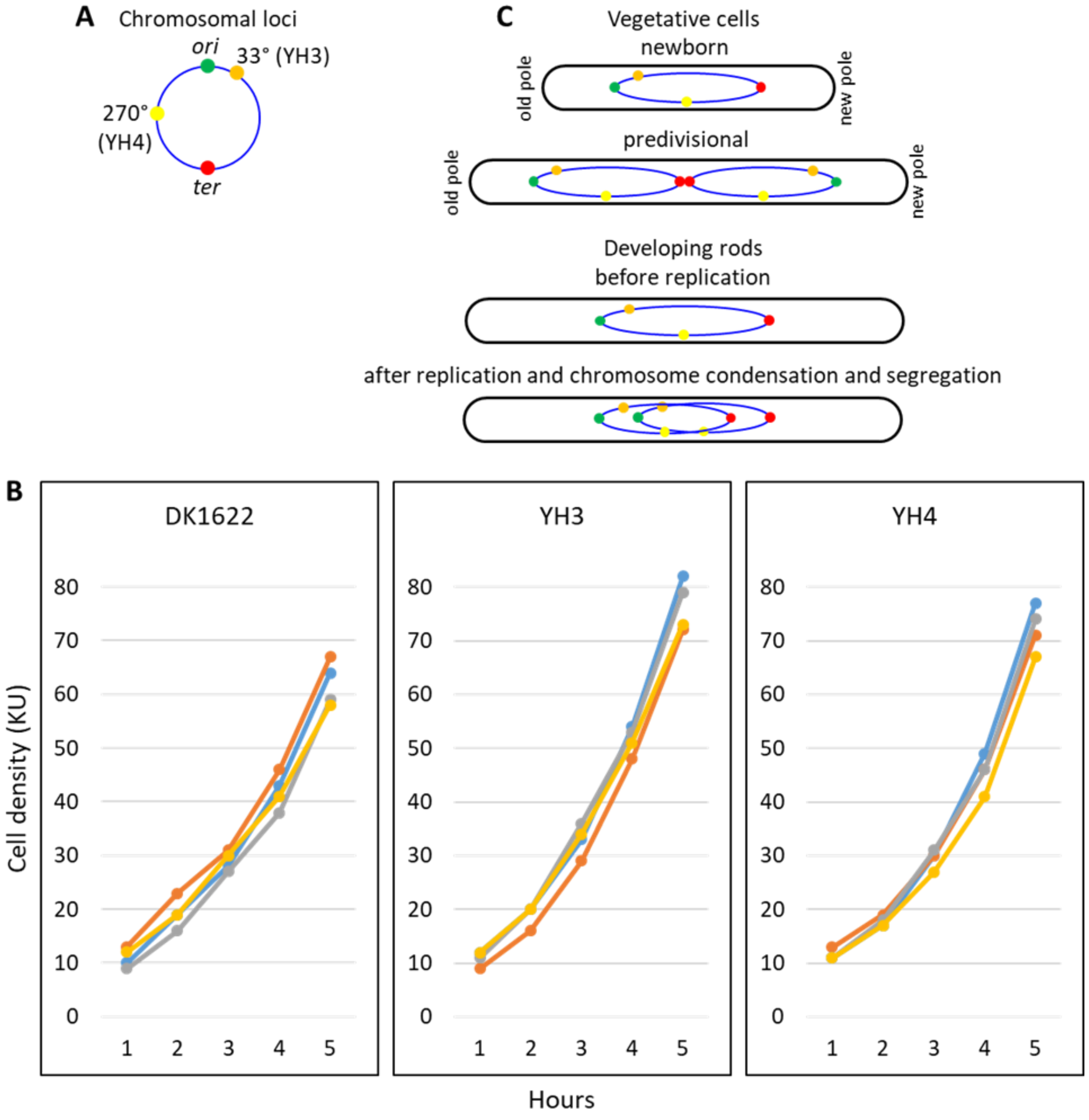

FIG S1 Locations of chromosomal loci and growth curves *M. xanthus* strains, and arrangement of chromosomes. (A) Locations of chromosomal loci. The locations of the *tetO*-arrays in strains YH3 and YH4 are shown relative to the origin (*ori*) of DNA replication and the terminus (*ter*) region. (B) Growth curves with or without IPTG addition. Exponentially growing cultures of wild-type strain DK1622, YH3, and YH4, were diluted to approximately equivalent cell density of 10 Klett units (KU). Each culture was divided and growth continued with or without IPTG (1 mM) addition. Cell density was measured at hourly intervals. For each strain, two biological replicates were performed. Orange and blue indicate one replicate with or without IPTG, respectively, and yellow and gray indicate the other replicate with or without IPTG, respectively. (C) Arrangement of chromosomes in vegetative cells and possibly in developing rods. Cartoons show the chromosome(s) in ~4 μm newborn and ~6 μm predivisional cells [3] with loci colored as in panel A, and plausible arrangements before and after replication in ~6 μm developing rods, assuming an average length of ~2.3 μm total for the nucleoid(s) (Fig. S6B) and an average distance between TetR-YFP foci of ~0.75 μm after replication (Fig. 1C).

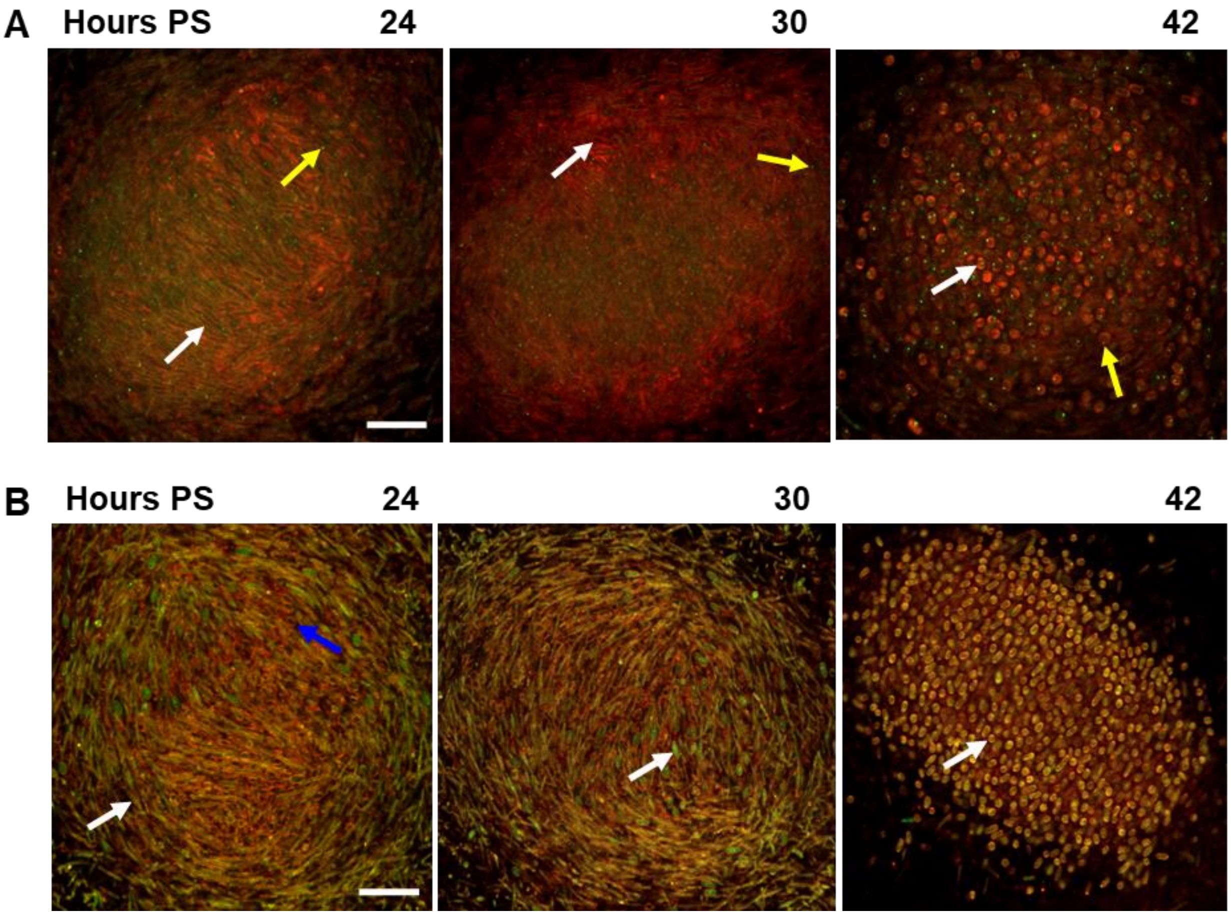

FIG S2 Visualization of chromosomal arrangement using a fluorescent repressor operator system during development. See the Figure 1 legend for a description of the experiment. Images of (A) YH4 and (B) YH5 show an optical section near the base of the same nascent fruiting body over time, with the yellow green (TetR-YFP) and red (FM 4-64 staining of the cellular membrane) channels merged. White arrows indicate a rod-shaped cell at 24 h poststarvation (PS), a transitioning cell at 30 h, and a spore at 42 h. Yellow arrows point to TetR-YFP foci. The blue arrow indicates a round cell lacking cytoplasmic fluorescence. Bars, 20 µm.

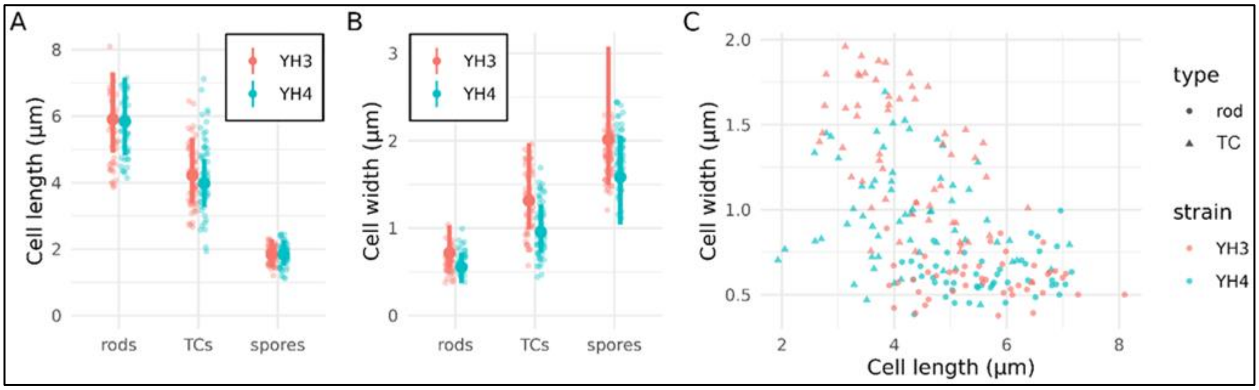

FIG S3 Dimensions of developing cells. In addition to measuring the distance between TetR-YFP foci for cells of strains YH3 and YH4 (shown in Fig. 1C), the length and width of rods and transitioning cells (TCs) was measured, and for spores, which are nearly spherical, the diameter was measured. Length (A) and width (B) of rods and TCs, and diameter of spores (same diameters are plotted in both panels, but note the different *y*-axes). The graphs show the measurements of individual cells (small dots), the median (large dot), and the 95% credible interval (vertical line). See Figure 1C for the numbers of cells analyzed. (C) Length and width of individual rods (dots) and TCs (triangles).

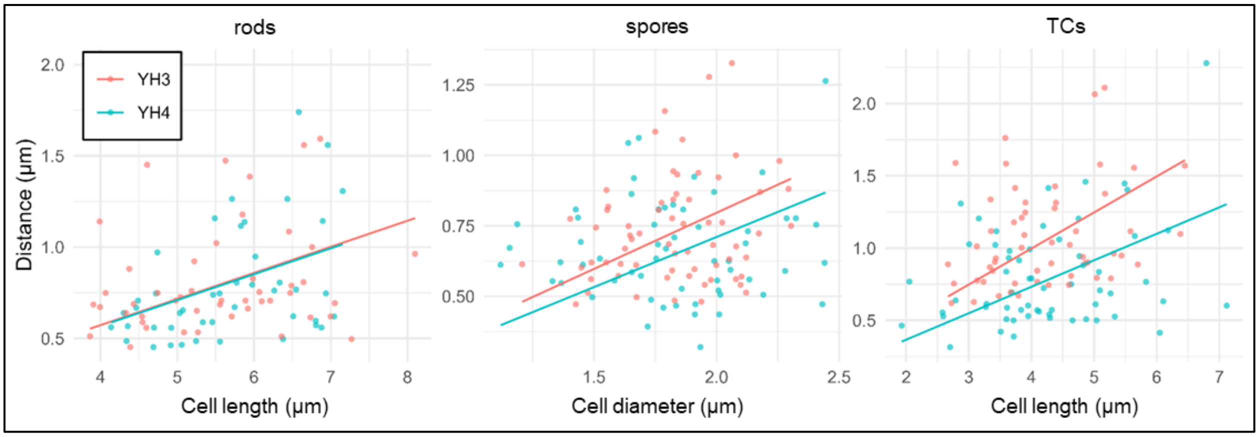

FIG S4 Distance between TetR-YFP foci compared with cell length or diameter. The distance between TetR-YFP foci for cells of strains YH3 and YH4 (shown in Fig. 1C) is plotted against the length of rods and transitioning cells (TCs), and against the diameter of spores (shown in Fig. S3A). The graphs show the measurements of individual cells (small dots) and best-fit lines for each strain. See Figure 1C for the numbers of cells analyzed.

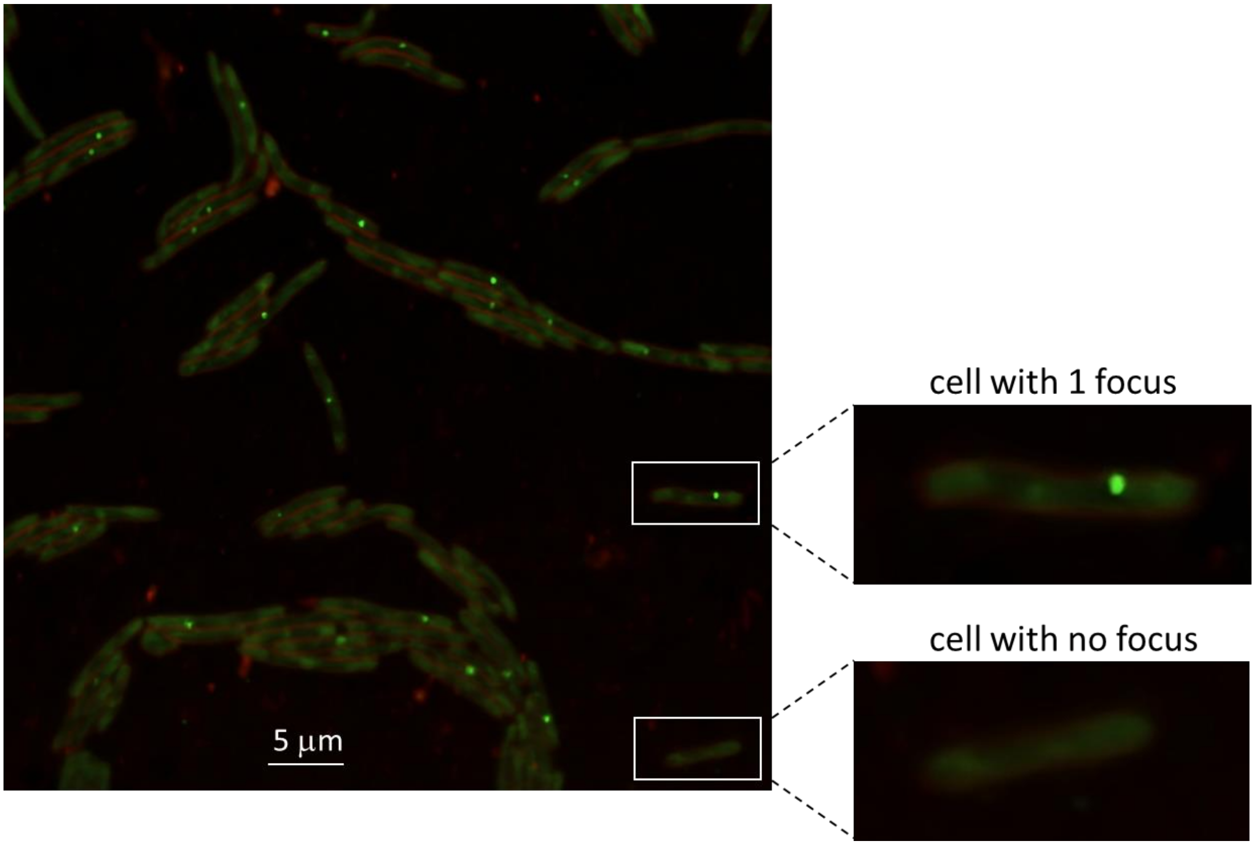

FIG S5 Visualization of TetR-YFP foci in vegetative cells. Exponentially growing cultures of strain YH4 containing a fluorescent repressor operator system were induced with IPTG (1 mM) for 4 h, cells were sedimented and resuspended in buffer containing IPTG (1 mM) and FM4-64 (5 μg/mL), and cells were visualized between an agarose pad and a glass cover slip. At left is a representative confocal image from two biological replicates, showing both TetR-YFP (yellow-green) and FM 4-64 staining of the cellular membrane (red). Cells appeared to be in a monolayer, with most adhered side-to-side in groups. White boxes indicate single cells with or without one TetR-YFP focus, and the boxed parts of the image are enlarged threefold at right.

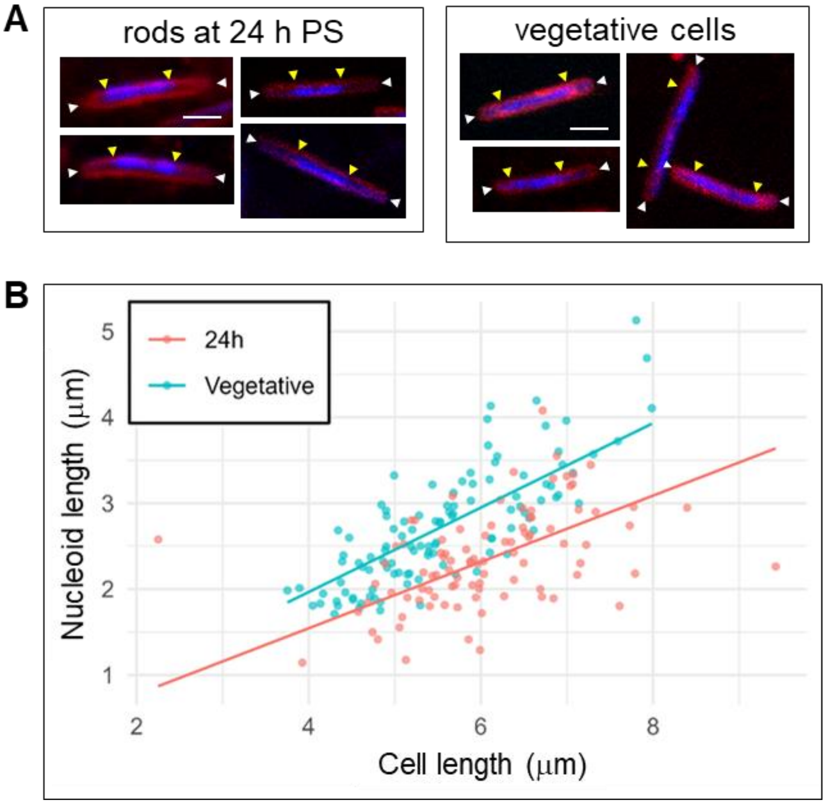

FIG S6 Nucleoid length compared with cell length for 24-h developing rods and vegetative cells. (A) Enlarged images of representative rods at 24 h poststarvation (PS) and during vegetative growth. For visualization of rods near the base of NFBs at 24 h PS, see the Figure 2 legend for a description of the experiment. For visualization of vegetative cells, exponentially growing cultures of wild-type strain DK1622 were treated with FM 4-64 (5 µg/mL) and DAPI (10 µg/mL) for 30 min before imaging in a monolayer between an agarose pad and a glass cover slip. Confocal images show FM 4-64 staining of the cellular membrane (red) and DAPI staining of DNA (blue) merged. White arrowheads point to the ends of cells and yellow arrowheads point to the ends of nucleoids. Bars, 2 µm. (B) Cell length and nucleoid length of rods at 24 h PS and during vegetative growth. The graph shows the measurements of individual cells (small dots) and best-fit lines for 24-h rods (total of 94) and vegetative cells (total of 109) from six biological replicates of each.

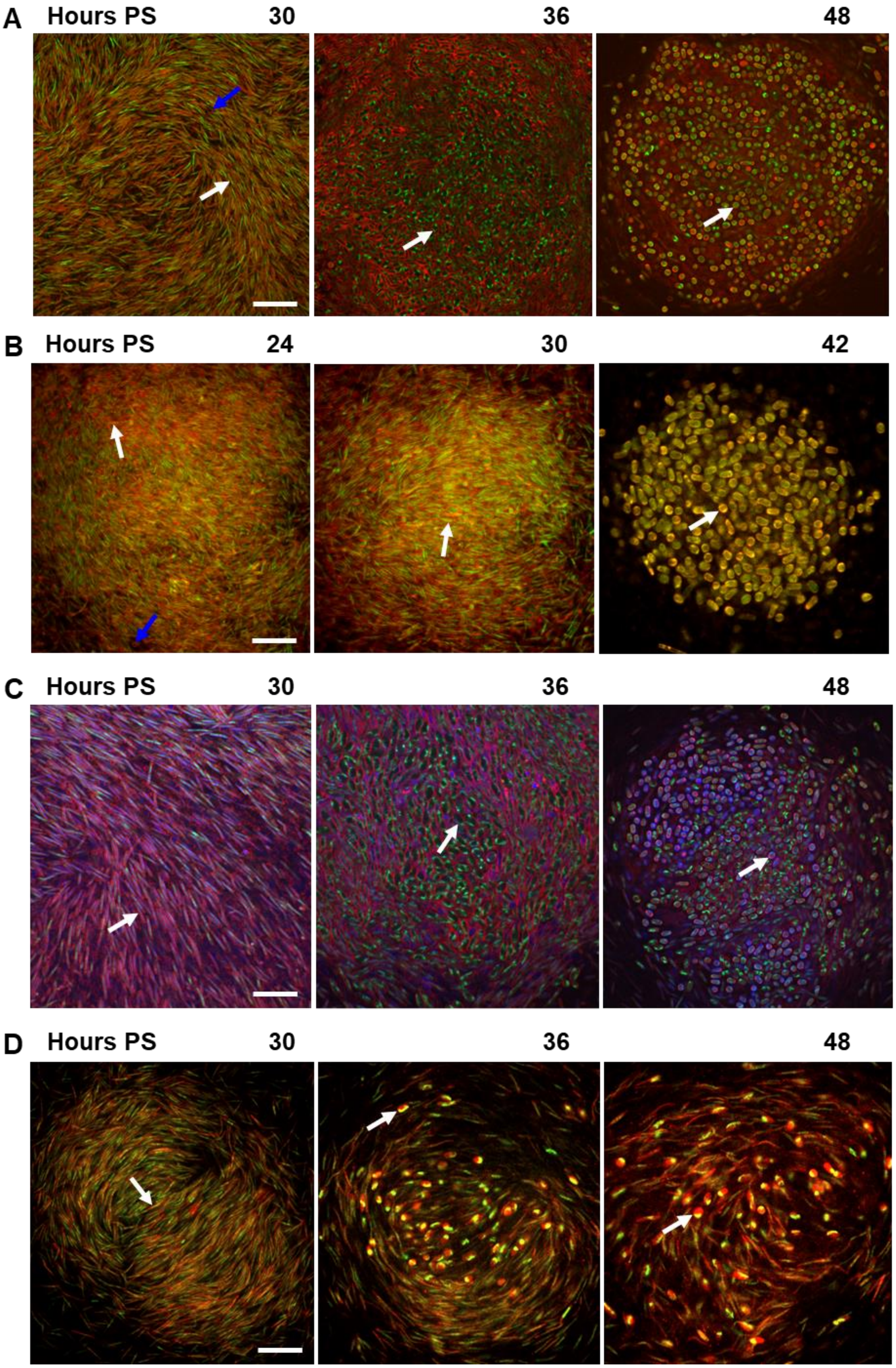

A Hours PS 30 36 48
B Hours PS 24 30 42
C Hours PS 30 36 48
D Hours PS 30 36 48

FIG S7 Visualization of nucleoids using mNeonGreen-FruA during development. *M. xanthus* strains were starved under submerged culture conditions. FM 4-64 (5 µg/mL) was added at the start of starvation unless indicated otherwise. Confocal images were acquired at the indicated times poststarvation (PS) and show FM 4-64 staining of the cellular membrane (red) and mNeonGreen-FruA fluorescence (green) unless indicated otherwise. (A) Images of strain YH6 alone (i.e., not mixed with wild-type strain DK1622) show an optical section near the base of the same nascent fruiting body (NFB) over time, with the red and green channels merged. White arrows indicate a rod-shaped cell at 30 h, a transitioning cell (TC) at 36 h, and a spore at 48 h. Blue arrow, round cell lacking cytoplasmic fluorescence. Bar, 20 µm. (B) Images of strain YH7 alone, which was engineered to produce mNeonGreen (not fused to FruA) as a control. YH7 was treated with vanillate (0.5 mM) (in addition to FM 4-64) at the start of starvation. Images show an optical section near the base of the same NFB over time, with the red and green channels merged. White arrows indicate a rod-shaped cell at 24 h, a TC at 30 h, and a spore at 42 h. Blue arrow, round cell lacking cytoplasmic fluorescence. Bar, 20 µm. (C) Images of representative NFBs of strain YH6 alone. DAPI (10 µg/mL) was added 30 min before imaging. Images show an optical section near the base of different NFBs, with the blue (DAPI), red, and green channels merged. White arrows indicate a rod-shaped cell at 30 h, a TC at 36 h, and a spore at 48 h. Bar, 20 µm. (D) Images of strain YH9 alone. Vanillate (0.5 mM) was added at the start of starvation to induce tdTomato production (cytoplasmic red fluorescence). FM 4-64 was not added. Images show an optical section near the base of the same NFB over time, with the red and green channels merged. Arrows indicate a rod-shaped cell at 30 h, a TC at 36 h, and a spore at 48 h. Bar, 20 µm.

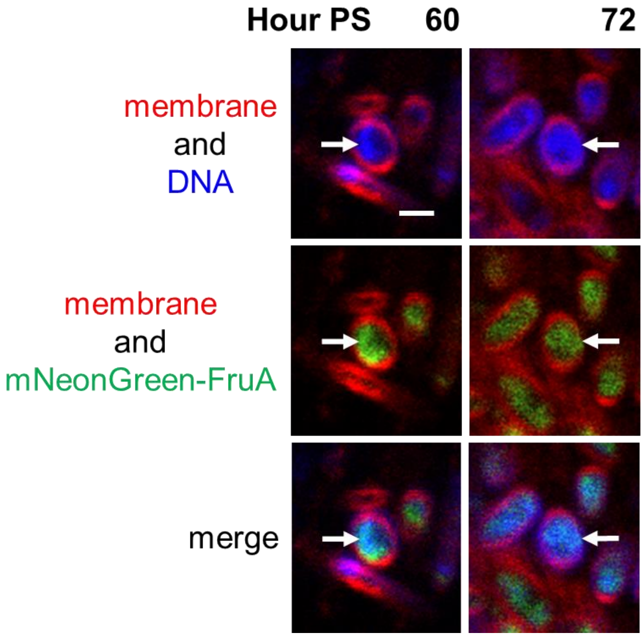

FIG S8 Visualization of nucleoids late in development. See the Figure 4A legend for a description of the experiment. Enlarged images of representative cells show spores at 60 and 72 h poststarvation (PS) with apparent partial and complete decondensation of nucleoids, respectively (arrows). Bar, 1 μm.

References

1. Hanahan, D., *Studies on transformation of Escherichia coli with plasmids.* J. Mol. Biol., 1983. **166**: p. 557-580.
2. Kaiser, D., *Social gliding is correlated with the presence of pili in Myxococcus xanthus.* Proc. Natl. Acad. Sci. USA, 1979. **76**: p. 5952-5956.
3. Harms, A., et al., *Tracking of chromosome and replisome dynamics in Myxococcus xanthus reveals a novel chromosome arrangement.* PLoS Genet., 2013. **9**(9): p. e1003802.
4. Hoang, Y., et al., *Cell density, alignment, and orientation correlate with C-signal-dependent gene expression during Myxococcus xanthus development.* Proc. Natl. Acad. Sci. USA, 2021. **118**(45): p. e2111706118.
5. Iniesta, A.A., et al., *Two systems for conditional gene expression in Myxococcus xanthus inducible by isopropyl-β-D-thiogalactopyranoside or vanillate.* J. Bacteriol., 2012. **194**(21): p. 5875-5885.
6. Lau, I.F., et al., *Spatial and temporal organization of replicating Escherichia coli chromosomes.* Mol. Microbiol., 2003. **49**(3): p. 731-743.
7. Julien, B., A.D. Kaiser, and A. Garza, *Spatial control of cell differentiation in Myxococcus xanthus.* Proc. Natl. Acad. Sci. USA, 2000. **97**(16): p. 9098-9103.
